# Endosperm structure and Glycemic Index of Japonica Italian rice varieties

**DOI:** 10.3389/fpls.2023.1303771

**Published:** 2024-01-05

**Authors:** Filip Haxhari, Francesco Savorani, Mariangela Rondanelli, Enrico Cantaluppi, Luigi Campanini, Edoardo Magnani, Cinzia Simonelli, Gentian Gavoci, Alessandro Chiadò, Mattia Sozzi, Nicola Cavallini, Angelica Chiodoni, Clara Gasparri, Gaetan Claude Barrile, Alessandro Cavioni, Francesca Mansueto, Giuseppe Mazzola, Alessia Moroni, Zaira Patelli, Martina Pirola, Alice Tartara, Davide Guido, Simone Perna, Roberto Magnaghi

**Affiliations:** ^1^ Centro Ricerche sul Riso, Ente Nazionale Risi, Castello D’Agogna, Italy; ^2^ Department of Applied Science and Technology (DISAT), Polytechnic University of Turin, Torino, Italy; ^3^ Department of Public Health, Experimental and Forensic Medicine, University of Pavia, Pavia, Italy; ^4^ Center for Sustainable Future Technologies @Polito, Istituto Italiano di Tecnologia, Torino, Italy; ^5^ Endocrinology and Nutrition Unit, Azienda di Servizi alla Persona ‘‘Istituto Santa Margherita’’, University of Pavia, Pavia, Italy; ^6^ Fondazione Policlinico Universitario Agostino Gemelli, Istituto di Ricovero e Cura a Carattere Scientifico (IRCCS), Rome, Italy; ^7^ Department of Biology, College of Science, University of Bahrain, Zallaq, Bahrain; ^8^ Sede Centrale, Ente Nazionale Risi, Milano, Italy

**Keywords:** Glycemic Index, diabetes, endosperm, scanning electron microscopy, starch granule, amyloplast, Japonica rice

## Abstract

**Introduction:**

Given that rice serves as a crucial staple food for a significant portion of the global population and with the increasing number of individuals being diagnosed with diabetes, a primary objective in genetic improvement is to identify and cultivate low Glycemic Index (GI) varieties. This must be done while ensuring the preservation of grain quality.

**Methods:**

25 Italian rice genotypes were characterized calculating their GI “in vivo” and, together with other 29 Italian and non-Italian genotypes they were studied to evaluate the grain inner structure through Field Emission Scanning Electron Microscopy (FESEM) technique. Using an ad-hoc developed algorithm, morphological features were extracted from the FESEM images, to be then inspected by means of multivariate data analysis methods.

**Results and Discussion:**

Large variability was observed in GI values (49 to 92 with respect to glucose), as well as in endosperm morphological features. According to the percentage of porosity is possible to distinguish approximately among rice varieties having a crystalline grain (< 1.7%), those intended for the preparation of risotto (> 5%), and a third group having intermediate characteristics. Waxy rice varieties were not united by a certain porosity level, but they shared a low starch granules eccentricity. With reference to morphological features, rice varieties with low GI (<55) seem to be characterized by large starch granules and low porosity values. Our data testify the wide variability of Italian rice cultivation giving interesting information for future breeding programs, finding that the structure of the endosperm can be regarded as a specific characteristic of each variety.

## 1 Introduction

The concept of Glycemic Index (GI), introduced in 1981 (Jenkins et al., 1981), allows to classify foods according to their postprandial glycemic response with reference to a carbohydrate source whose value is considered equal to 100 (Foster-Powell et al., 2008). GI values are hence conventionally expressed as a percentage compared to glucose or white bread (ISO 26642) (International Standards Organization, 2010), the latter having a GI of 70 compared to the former (Hallfrisch and Behall, 2000).

Several studies revealed a correlation between the consumption of high-GI foods, causing a rapid rise in blood-glucose levels, and Type 2 Diabetes (Livesey et al., 2013; Bhupathiraju et al., 2014; Yao et al., 2023), cardiovascular diseases (Sheu et al., 2011; Ma et al., 2012; Mirrahimi et al., 2012), and cancer (Choi et al., 2012; Turati et al., 2015).

In Italy, there are about 3.5 million people with diabetes, accounting for 5.8% of the entire population (Istat, “Report anni 2000-2016 Il diabete in Italia”, 2017). Instead, according to the International Diabetes Federation, the number of diabetic subjects between the ages of 20 and 79 years in Europe is about 61.4 million, or 9.2 percent (“IDF Diabetes Atlas, 10th edition”, 2021).

Rice is the most important staple food, especially in low- and middle-income countries, where it provides 27% of the diet calories, representing the main source of livelihood for nearly 4 billion people worldwide. It is also estimated that global demand for rice is likely to continue to increase to overcome 536 million tons (milled rice) by 2030 (CGIAR research program on Rice_ Importance of Rice, https://ricecrp.org/importance-of-rice/, accessed on 30th 11 2022). In 2020 rice was cultivated on about 164.2 million hectares (corresponding to 22.3% of the total cereals area and 11.8% of world’s arable land), producing 756.7 million tons paddy rice (nearly 25.3% of the whole cereal production) (FAOSTAT. https://www.fao.org/faostat/en/#data, accessed on 05th 12 2022).

In view of the increasing number of people affected by diabetes all over the world and of the important role that a such a widely consumed and valued staple food could play in enhancing human nutrition (Dwiningsih and Alkahtani, 2023; Srikaeo, 2023), a careful diet based on low-GI rice varieties could represent a successful strategy to prevent diabetes and reduce the impact of other metabolic disorders, halting the progression of prediabetes and preventing the development of hyperglycemia and its related complications, such as cardiovascular diseases (Abdullah et al., 2009; Blaak et al., 2012), thus improving the health condition of affected subjects. It is therefore of paramount importance to identify and develop rice varieties characterized by a low GI, without neglecting grain quality and its organoleptic characteristics to better meet the needs of consumers.

Rice, which is rich in carbohydrates and easily digestible, is often included among high-GI foods (Goñi et al., 1997; Atkinson et al., 2008; Lovegrove et al., 2019), thus marking it as not particularly suitable for people suffering from diabetes or other metabolic disorders. This is not always true as the GI values of rice are indeed highly variable (Hallfrisch and Behall, 2000; Howlader and Biswas, 2009; Fitzgerald et al., 2011; Chiu and Stewart, 2013; Dwiningsih and Alkahtani, 2023).

Many factors, such as cooking method and time, condiments and accompaniments, presence of antioxidant components (phenolic compounds in pigmented varieties), degree of polishing of the grain and industrial treatments, can affect rice GI (Hallfrisch and Behall, 2000; Larsen et al., 2000; Hettiarachchi et al., 2001; Sun et al., 2010; Boers et al., 2015; Dwiningsih and Alkahtani, 2023; Ngo et al., 2023; Srikaeo, 2023; Yao et al., 2023); however, comparing different varieties under the same conditions also reveals the existence of a strong genetic component. A wide study conducted by the International Rice Research Institute (IRRI) and the Commonwealth Scientific and Industrial Research Organisation’s (CSIRO) Food Futures Flagship on 235 rice varieties from all over the world, highlighted a great variability in GI, with values between 48 and 92 (average value 64), compared to glucose (100) (Fitzgerald et al., 2011).

The genetic basis of GI in rice is complex and involves multiple factors (Rathinasabapathi et al., 2015), thus increasing the difficulties in performing breeding programs aimed to develop low-GI varieties, more suitable for diabetic subjects. Among the multiple factors involved in determining rice GI, the Waxy gene (*Wx*) has been suggested to play a main role (Fitzgerald et al., 2011).

Starch represents the main form of energy storage in plants, as well as the main component of rice grain endosperm, and has a key role in determining rice postprandial glycemic response, and consequently its GI.

Rice starch is composed by two different polymers: amylose, a relatively short polymer of glucose units linked by α 1→4 bonds that is characterized by a mainly linear structure, and amylopectin, a longer polymer where glucose units are arranged linearly by α 1→6 bonds occurring every 24 to 30 glucose units, that is characterized by a branched structure (Hallfrisch and Behall, 2000; Vandeputte and Delcour, 2004; Matsushima et al., 2010; Rathinasabapathi et al., 2015). Actually, rice amylose comprises a mixture of linear and branched molecules (up to the 39%), albeit to a decidedly lesser extent than amylopectin (55-60%); the number of chains of branched amylose is variable, so that its structure is a characteristic of each variety (Juliano, 1992).

Differing from other main cereals, rice endosperm is characterized by the presence of compound starch granules, which are usually 10–20 µm in diameter (Matsushima, 2015). Each of them consists of many smaller single granules with an average size between 2–3 and 7–10 µm, as reported in several publications (Juliano, 1992; Jane et al., 1994; Lindeboom et al., 2004; Yun and Kawagoe, 2009; Matsushima, 2015; Patindol et al., 2015).

Amylose content in rice cultivars is greatly variable, resulting practically absent in glutinous or “waxy” varieties and reaching 30% or even more in some non-waxy ones and up to 40% in amylose extender mutants (Kongseree and Juliano, 1972; Juliano, 1992; Miller et al., 1992; Boers et al., 2015; Patindol et al., 2015). Rice varieties can then be classified according to their amylose content as follows: waxy (0-2%), very low (2-12%), low (12-20%), medium (20-25%) and high (25-33%) (Juliano, 1993).

Varieties having a high amylose content usually show a lower GI, therefore the correlation observed between the two factors suggests that amylose represents the most determining component for rice GI, although not the only one (Panlasigui et al., 1991; Indrasari et al., 2010; Fitzgerald et al., 2011; Ngo et al., 2023).

The qualitative characteristics of the product (grain) are of great importance to consumers, considering that rice is mainly consumed as a whole grain instead of flour, but requirements differ according to geographical area and local culinary traditions. Breeding programs should therefore consider the characteristics of the grains in relation to the intended use of each type or variety of rice.

More than 170 rice varieties are currently grown in Italy, belonging to five main commercial groups (Round, Medium, Long A for national market, Long A for parboilization and Long B) and differing in grain shape and length, but also in grain inner structure (showing a chalky fraction of the grain, in a variable ratio, or having a more compact and vitreous texture) and in amylose content. Italian rice is thus characterized by great variability, which has never been studied in depth.

According to our knowledge, no studies were published regarding GI and grain physical structure of a conspicuous number of Italian or European rice varieties. In this article we present data about “*in vivo*” GI evaluation of 25 Italian rice genotypes, and concerning the morphological characterization of endosperm inner structure of 36 Italian genotypes and other 18 non-Italian rice varieties for a total number of 54 genotypes. Those data could represent the starting point for future breeding programs aimed to develop rice lines more suitable for diabetic consumers, having also the most appropriate characteristics for each destination of use.

## 2 Materials and methods

### 2.1 Plant material and processing information

This study evaluated a total of 54 rice varieties (*Oryza sativa*) from different points of view (GI, biochemical traits, inner structure of the grain). Among them 36 were Italian (**Table 1**) and 18 of non-Italian origin (**Table 2**). All the Italian varieties considered in this study were chosen among those of which the Ente Nazionale Risi is the breeder and/or maintainer. We preferred to characterize only Ente Nazionale Risi varieties because almost all the other varieties registered in the National Register of Varieties of Agricultural and Horticultural Species, or cultivated in Italy, belong to private seed companies, holding their rights.

**Table 1:** Italian rice varieties and advanced lines provided by Ente Nazionale Risi.

Denomination	Grain type
Arborio	Long A national market (risotto)
Argo	Medium
Baldo	Long A national market (risotto)
Carnaroli	Long A national market (risotto)
Castelmochi	Round (waxy)
CL 71	Long B
CL12	Round
CL18	Round
CL31	Long A for parboilization
CL35	Long A for parboilization
CL388	Long A national market (risotto)
CL510	Long A national market (risotto)
CL80	Long B
Cripto	Medium
CRLB1	Long B
CRW3	Round (waxy)
Dedalo	Long B
Drago	Long A for parboilization
Duilio	Medium
Elio	Round
Enr-18126	Long B
Enr-18215	Round
Enr-18328	Long B
Enr-18433	Long A for parboilization
Europa	Long A for parboilization
Iarim	Long B
Italmochi	Medium (waxy)
Lince	Long A for parboilization
Padano	Medium
Pegaso	Long B
Prometeo	Round
Puma	Long A for parboilization
S. Andrea	Long A national market (risotto)
Selenio	Round
Tiberio	Long A for parboilization
Valente	Long A for parboilization

**Table 2:** Rice germplasm provided by International Rice Research Institute. (https://gringlobal.irri.org/gringlobal/search.aspx).

Denomination	Genotype ID
Cisokan	IRGC 76981
Cypress	IRGC 117282
Doongara	IRGC 78392
Fedearroz 50	IRGC 117395
Hetadawee	IRGC 12084
Iac 165	IRGC 82775
IR 42	IRGC 39291
IR 4630-22-2-5-1-3	IRGC 72958
IR 50	IRGC 53433
IR 6	IRGC 51504
IR 64	IRGC 66970
Kahawanu	IRGC 36263
Kaluheenati	IRGC 7750
Mahsuri	IRGC 10929
Pajam	IRGC 25910
Sinandomeng	IRGC 78627
Swarna	IRGC 122258
Taichung Sen 17	IRGC 78210

The number of varieties considered (**Table 1**) is therefore not particularly high compared to those existing/on the market, but they were chosen to represent the different product groups, including both varieties that are still cultivated on the national territory and varieties currently no longer in cultivation, and also some recently selected lines: the result of the genetic improvement activity carried out at the institute’s Research Centre. The list of the Italian varieties considered includes both recent and traditional varieties, with different types of grain (rice with completely crystalline or partially chalky grains, waxy rice, aromatic rice) and belonging to different product groups: Round grain, Medium grain, Long A grain for the National market (risotto), Long A grain for parboilization and Long B grain, with the aim of representing almost all the types present on the Italian market.

Many famous and appreciated Italian traditional cultivars, such as: Arborio, Baldo, Carnaroli and S.Andrea, considered among the most suitable for the preparation of risottos, were also included in this study.

As regards non-Italian varieties, the list of those considered was dictate by two main factors: choosing varieties whose GI was already known and reported in literature and which the International Rice Research Institute (IRRI), situated in Los Baños, Philippines, could provide us for research purposes. To be sure that the genotypes considered, mainly represented by Asian landraces or lines developed by IRRI itself, were exactly those whose GI values were reported in literature, we chose to use only genotypes whose ID (**Table 2**) was reported in the corresponding scientific publications.

Those considered in this study are therefore in most cases well-known varieties, already registered and cultivated, and for this reason they present characteristics of distinguishability, uniformity and stability and show recognizable and hereditary traits, which means that they are genetically different.

The number of rice varieties to study was determined based on the original objective of characterizing the highest number of rice varieties belonging to the different product groups as possible, to deepen their knowledge, with the further aim of implementing genetic improvement programs, compatible with time and resources limitations.

To the best of the authors’ knowledge, this is one of the few studies on these topics considering a such large pool of European and Italian varieties.

All the rice samples analyzed in this study were produced in 2020 at the Rice Research Center of Ente Nazionale Risi, located in Castello D’Agogna (27030, PV, Italy; 45°14 ‘53’’N, 8°42’00’’E), using “Basic” category seed for sowing. Paddy rice samples were harvested manually at the end of the season and dried until the storage humidity, below 14%, was reached. Samples produced in the same year and in the same place were used, to minimize possible environmental effects, and thus bring out the variability linked to the differences among varieties.

Biochemical analyzes and GI evaluation were carried out on 25 Italian rice genotypes, deemed the most interesting and promising among the 36 considered (**Table 3**), using white rice samples processed at the Rice Research Center employing a “Marumasu Pelicano” dehuller and a continuous whitening machine. After processing, broken grains were removed from all samples using a “Satake TRG rice length grader” laboratory machine. The white rice samples thus obtained showed high uniformity both in milling degree and grain size, and were therefore comparable to commonly marketed rice available to the final consumer. For each variety subjected to GI evaluation, 10 kg of milled rice were produced.

**Table 3:** Glycemic Index (GI) evaluation and characterization of 25 Italian rice genotypes through Field Emission Scanning Electron Microscopy (FESEM) imaging.

Denomination	Average starch granules size (area) ± SD (*μ*m^2^)	Starch granules average eccentricity	Starch granules average circularity	Estimated diameter of starch granules (μm)	Average porosity (%)	Protein content milled rice (g/100 g)	Ashes (g/100 g)	Total fat substances milled rice (g/100 g)	Amylose content (%)	Average GI milled rice (% glucose)
**High Glycemic Index**										
Arborio	43.02±5.13	0.71	0.40	7.40	5.79±0.27	6.71±0.41	0.43±0.04	0.57±0.05	14.10±3.00	92.31±8.35
Lince	123.20±15.59	0.69	0.42	12.53	1.11±0.09	5.97±0.37	0.34±0.04	0.64±0.05	17.50±3.50	88.93±9.22
Duilio	34.90±6.50	0.73	0.37	6.67	4.12±3.05	7.78±0.48	0.60±0.05	0.78±0.06	9.60±2.30	86.22±10.18
Castelmochi					0.89±0.23	7.54±0.46	0.66±0.05	1.44±0.09	< 5	84.71±10.65
Padano	36.40±2.26	0.73	0.40	6.81	6.55±0.05	6.69±0.41	0.60±0.05	0.95±0.07	12.70±2.80	73.69±10.66
CL18	93.41±6.51			10.91	0.63±0.46	6.83±0.42	0.53±0.05	0.81±0.06	11.60±2.60	73.01±8.17
Puma	64.62±44.32	0.72	0.39	9.07	2.83±3.04	5.64±0.35	0.61±0.05	0.69±0.05	14.00±3.00	73.00±7.05
Baldo	74.90±24.86	0.72	0.40	9.77	3.60±2.91	7.28±0.45	0.48±0.04	0.78±0.06	14.20±3.00	71.42±6.44
CL 71	39.46±26.27	0.73	0.40	7.09	3.30±2.47	7.43±0.46	0.58±0.05	0.94±0.07	20.70±4.00	71.29±7.14
CL35	39.35±2.12			7.08	6.49±0.43	5.79±0.36	0.60±0.05	0.99±0.07	14.40±3.00	71.03±9.76
**Medium Glycemic Index**										
CL12	42.55±8.74			7.36	3.48±4.50	6.78±0.42	0.54±0.05	0.94±0.07	13.00±2.80	68.69±7.03
S. Andrea	38.06±6.42	0.73	0.39	6.96	5.95±0.28	5.93±0.37	0.51±0.05	0.74±0.06	15.20±3.10	66.49±7.64
Valente		0.73	0.38		0.69±0.59	7.18±0.44	1.08±0.07	0.75±0.06	12.10±2.70	66.17±6.86
Carnaroli	36.85±6.54	0.70	0.40	6.85	5.89±0.27	6.63±0.41	0.40±0.04	0.75±0.06	20.70±4.00	64.17±6.50
CL388	39.59±8.18	0.72	0.40	7.10	5.53±1.40	7.43±0.46	0.41±0.04	0.63±0.05	12.60±2.70	62.56±8.67
Tiberio	52.58±37.36	0.73	0.42	8.18	3.07±2.24	6.66±0.41	0.58±0.05	0.91±0.06	23.90±4.50	61.77±5.99
CRLB1	49.11±4.30	0.72	0.42	7.91	0.83±0.16	7.58±0.46	0.53±0.05	0.71±0.05	21.80±4.10	61.06±3.73
Elio	44.48±5.99	0.72	0.39	7.53	6.42±0.44	5.98±0.37	0.41±0.04	0.76±0.06	22.90±4.30	60.39±5.87
Enr-18126	68.57±27.12	0.72	0.38	9.35	3.40±2.93	7.34±0.45	0.22±0.04	0.70±0.05	17.70±3.50	58.45±5.83
Iarim	55.43±30.14	0.74	0.38	8.40	3.83±2.30	7.36±0.45	0.59±0.05	0.92±0.06	24.40±4.50	58.00±9.29
**Low Glycemic Index**										
Enr-18215		0.73	0.40		0.77±0.25	6.69±0.41	0.46±0.04	0.88±0.06	12.00±2.70	54.26±6.79
Enr-18328	90.62±12.61	0.73	0.40	10.74	0.53±0.26	6.91±0.42	0.35±0.04	0.77±0.06	23.40±4.40	53.56±5.01
Argo	77.87±7.50	0.71	0.41	9.96	6.72±0.39	7.37±0.45	0.46±0.04	1.08±0.07	20.30±3.90	50.55±7.17
Enr-18433		0.72	0.40		0.44±0.22	6.09±0.37	0.56±0.05	0.87±0.06	18.70±3.70	49.21±5.59
Selenio	107.77±24.93	0.71	0.43		0.40±0.28	6.33±0.39	0.21±0.04	0.69±0.05	14.60±3.00	49.15±6.55

Values ± standard deviation are reported. Missing values are due to the particularly compact nature of the starch granules, which the algorithm could not properly process.

A small aliquot of the rice samples was sent to the Chelab laboratory in Resana (TV, Italy) to carry out biochemical analyzes and another was stored at the Rice Research Center for amylose content quantification.

The study on the inner structure of the grain, carried out by field emission scanning electron microscopy (FESEM) on paddy rice samples, involved 54 genotypes: 36 Italian varieties and selected lines, i.e. the 25 used in the study on GI and, in addition to them, 11 other genotypes (**Table 1**), together with 18 genotypes belonging to the IRRI genebank (**Table 2**). Paddy rice grains of each variety were carefully chosen to avoid distorted or damaged grains.

It should be noted that the term “paddy rice” refers to the rice grain wrapped in the husks, as it is harvested in the field. “Brown rice” refers to the rice grain without husks, obtained from paddy rice after the dehulling process, while the term “white rice” refers to rice grains subjected to the whitening process, and thus deprived of the germ and of the outermost layers of the grain which are rich in fibers, lipids and proteins; for this reason, milled rice consists of the endosperm of the grain and is composed almost exclusively of starch.

### 2.2 Glycemic Index evaluation

This study was designed based on the standard method for determining GI, published by International Standard Organization ISO 26642 (International Standards Organization, 2010). The GI was calculated evaluating the blood glucose response curve of 10 volunteers (Rondanelli et al., 2023). A glucose solution was used as reference food for GI evaluation.

Healthy, non-smoking volunteers were recruited from staff and students from June 11^th^, 2021 to March 1^st^, 2022, at Dietetic and Metabolic Unit of the Santa Margherita Institute, University of Pavia, Italy. Baseline characteristics of the participants were obtained. The inclusion criteria were healthy adults aged 18–40 with Body Mass Index (BMI) between ≥ 18.5 and ≤ 25 kg/m^2^. The exclusion criteria were fasting blood glucose (FBG) ≥ 5.5 mmol/L; presence of chronic diseases (cardiovascular diseases, diabetes type 1 and 2, hypertension, metabolic syndrome, cancer, renal disorders, digestive tract diseases, celiac disease); following a special diet; and taking any medication concerning glucose metabolism, thyroid function, any dietary supplements. Athletes, as well as pregnant or breast-feeding women, were also excluded from the study.

All participants gave written informed consent. The study was conducted according to the guidelines of the Declaration of Helsinki, and all procedures were approved by the Local Ethics Committee (ethical code number: 2608/11012022) and recorded on Clinicaltrials.gov (NCT05333081).

The participants were asked to attend the Dietetic and Metabolic Unit of the Santa Margherita Institute at 8:30 AM for 26 days: 25 days for food tests, and 1 day for reference food (glucose) with a 2-day washout period (International Standards Organization, 2010). Prior to being tested, the participants were instructed to fast for 12 hours the day before and avoid any vigorous activities, smoking, alcohol consumption, high-fiber foods, indigestible high-carbohydrates and high-fat/high carbohydrates (Wolever et al., 2008). Continuation of daily routines, such as physical activity and diet was encouraged.

Anthropometric parameters of the volunteers, such as weight and height, were measured by the same investigator (Frisancho, 1984). Body Mass Index (BMI) was calculated (kg/m^2^) prior to the day of the test.

White rice samples, of the considered 25 rice genotypes (**Table 3**), were provided by Ente Nazionale Risi to the laboratories of Dietetic and Metabolic Unit of the Santa Margherita Institute, University of Pavia, to carry out the analyses. The samples were stored at a controlled temperature of 4°C until their administration to the volunteers. Anhydrous glucose (Merck Co.), 50 g dissolved in 250 mL water at room temperature, was used as the reference food. Participants were instructed to consume the prepared reference carbohydrate solution over a period of 12 to 15 minutes. Food portions corresponding to 50 g of carbohydrates were administered to volunteers for each rice variety and for the reference food.

For the blood glucose response test, capillary blood samples were taken from the middle and ring fingers (Brouns et al., 2005; International Standards Organization, 2010). Initially, the participants were asked to wash their hands and then two finger-prick blood samples were taken at -5 and 0 minutes while fasted. Postprandial blood glucose was measured 15 minutes after the first bite and at 30, 45, 60, 90, and 120 minutes. This procedure is illustrated in **Supplementary Figure 1**. Blood glucose was measured in the whole blood using a reliable standard glucometer (ACCU-CHEK Performa-Roche Diagnostics GmbH, Germany) (Freckmann et al., 2012), calibrated with manufacturer’s control solutions 15 minutes before the test.

The inter-assay coefficient of variation (CV) on the standard solution was less than 3, and the laboratory’s CV for 24 duplication measurements of fasting glucose was less than 3.03%, both meeting usual standards, whereby CV should be lower than 3.6% and 5%, respectively (International Standards Organization, 2010).

The same food administration and blood sampling procedures adopted for the glucose solution were also used for the test foods. The order of food items tested was the same for all participants.

### 2.3 Glycemic Index calculation and statistical analysis

The GI value of the rice was calculated based on the method described by ISO 26642-2010 as the incremental area under the curve (IAUC) of a 50 g carbohydrate portion of the test food expressed as a percent of the response to the same amount of carbohydrate from a reference food taken by the same subject.

Blood glucose response was expressed as the incremental area under the blood glucose response curve and was calculated using the trapezoidal method and the IAUC method, based on a mathematical formula (disregarding the area below baseline). The Incremental Area Under blood glucose response Curve (IAUC) for each test food ingested by each subject was expressed as the percentage of the mean IAUC glucose for three repetitions of the reference food (glucose) consumed by the same subject, as GI = (IAUC test food/mean IAUC glucose) × 100. The mean values for all subjects were considered the GI of a given test food. The GI values were categorized into low, medium, or high glycemic response. The cut-off for GI values was: ≤ 55, 56 - 69, ≥ 70, respectively (Foster-Powell et al., 2008). Data were analyzed by SPSS version 22.0. A correlation matrix was created to verify the existence of a relationship between amylose content and GI. p < 0.05 was considered as significant.

Data were thus obtained following a widely shared and used methodology for estimating GI, whose protocol requires the determination of the glycemic curves in 10 healthy volunteers to then calculate GI based on the average values. Consequently, the volunteers themselves represent the replicates, according to this methodology.

### 2.4 Biochemical analysis

Biochemical analyses on the same rice milled samples used for GI evaluation were performed in outsourcing by an accredited laboratory (Chelab, Mérieux NutriSciences Corporation, Resana, Treviso province, Italy), according to the following methods:

- Protein content (g/100 g) MP 1457 rev 3 2017;
- Total fat substances (g/100 g) ISTISAN reports 1996/34 MET Pg. 41;
- Ashes (g/100 g) MP 2271 rev 0 2018;
- Carbohydrates (g/100 g) MP 0297 rev 6 2018;
- Energy value (kcal/100 g) MP 0297 rev 6 2018;

The apparent amylose content was determined by the accredited Merceological and Chemical Laboratory of Ente Nazionale Risi, situated in the Rice Research Centre (Castello D’Agogna, 27030 PV, Italy), using a spectrophotometric method with a defatting procedure by methanol 85%. Amylose content (g/100 g d.m.) was calculated by colorimetric method with iodine, using a standard curve plotted from absorbance of amylose/amylopectin standards (ISO 6647-1:2020) (International Standards Organization, 2020); potato amylose and waxy rice amylopectin were used for calibration solutions (**Supplementary Figure 2**). The analytical determinations were conducted in duplicate as indicated in ISO 6647-1:2020 protocol.

### 2.5 Characterization of the internal structure of the grain in 54 rice varieties by field emission scanning electron microscope imaging

The paddy rice samples of 54 different varieties were analyzed by FESEM to observe the characteristics of their internal structure, determining starch granules dimensions and the porosity of the structure, understood as the percentage of empty spaces (area among the starch granules). To this aim, the paddy rice samples of 36 varieties and selected lines (**Table 1**), together with 18 paddy rice samples of IRRI genotypes (**Table 2**), were prepared as follows.

Paddy rice grains were cut longitudinally (for the entire length) by incision of the surface with a scalpel. The halves of the grains were then fixed on a microscope stub with conductive tape and subsequently metallized with a thin layer of Pt by DC sputtering in Ar atmosphere (Q150T-ES, Quorun Technologies) with a sputtering current of 30 mA for 25 s.

A Zeiss SUPRA 40 (Zeiss SMT, Oberkoechen, Germany) FESEM, equipped with a detector for secondary electrons and a detector for backscattered electrons, was used to acquire the images. Micrographs were acquired with an accelerating voltage of 5 kV and a 30 µm opening using a secondary electron detector. After fine-tuning the sample preparation technique, 3 grains for each variety were analyzed to obtain 4 images for each grain (1 at 100X and 3 at 5000X magnification). The 5000X images were acquired in the most representative areas of the grain section, chosen after carefully observing the 100X images, specifically looking for regions in which the cut did not alter the starch granules structure, to better capture and describe the granules’ characteristics and arrangement.

### 2.6 Image analysis and processing

An *ad hoc* algorithm was developed for image analysis and processing, using the MATLAB software development environment. The algorithm allows the recognition of the areas represented by the starch granules and the gaps among them, distinguishing those that are actual empty spaces from those that are shadows created by the image capture perspective (**Supplementary Figure 3**). The average percentage of porosity of the grain was thus automatically calculated from the obtained images together with the relative standard deviation. The average size of the starch granules (μm^2^) was calculated based on the number of pixels of each granule, and also for this parameter the standard deviation was calculated.

To estimate the porosity percentage of the endosperm and the average area of the starch granules, the mean of the values obtained from the three 5000X images of each grain and of the 3 grains analyzed for each variety was calculated. Furthermore, since the chalky and crystalline fractions of the grain, presenting completely diverse morphological characteristics, have different extents, a weighted average was calculated for each variety, which considers the extent of the chalky part of the endosperm, as a percentage.

Morphological parameters of starch granules were extracted using this image analysis procedure (i.e., the *ad hoc* developed algorithm): eccentricity, circularity and perimeter of the granules were calculated, obtaining for each parameter an average value for each variety. The eccentricity value indicates how closely the starch granule approximates an ellipse, having a different shape from a perfect circle (value equal to 0 represents the circle, growing up to 1 for an increasingly elongated ellipse). Instead, the circularity value expresses how far the starch granule differs from being a perfect circle (a value equal to 1 represents a circle while values tending to 0 indicate a different shape). Starch granules diameters were estimated from their average dimensions (area), considering them approximately as round shaped.

From the previously described analytical techniques two different datasets were obtained: one gathers the different biochemical traits (carbohydrates, nitrogen compounds, proteins, total fatty compounds, raw lipids, ashes and energy content) belonging to the 25 Italian varieties, and the other collects the morphological features (mean eccentricity, mean perimeter, mean area, mean circularity and porosity percentage) extracted from the 54 varieties treated with the *ad hoc* image analysis algorithm. For nine varieties the algorithm was not able to extract the features related to starch granules, because of the compact nature of the internal structure and starch spatial disposition.

### 2.7 Statistical analysis: exploratory multivariate data analysis

An exploratory multivariate data analysis by principal component analysis (PCA) (Bro and Smilde, 2014) was performed using the PLS_Toolbox (operating under MATLAB environment, version 8.9, Eigenvector Research Inc., Manson, WA, USA) software package. PCA was applied to explore the information content of the biochemical data and other assessed characteristics of the studied varieties.

The biochemical and morphological data were imported into MATLAB to be pre-processed and inspected with PCA, which is a multivariate statistic approach able to highlight the correlations among the properties used to describe the data, and which also allows to explore the samples distribution (i.e., the different rice varieties), which can be explained according to the different properties, i.e., the variables. This technique performs a mathematical decomposition of the data table and creates a new set of variables, called Principal Components (PC), that represent the sources of maximum variance (a statistical quantity directly related to the amount of information). The results of this analysis allow to visualize the input data in a new set of coordinates identified as the “Principal Components space”. The exploration of this low-dimensional space provides the information related to the samples’ distribution as the so-called “scores plot”, and the variables’ distributions and correlations as the so-called “loadings plot”. The scores plots allow finding clusters of similar samples, trends related to specific properties and even possible outliers. The loadings plots allow inspecting the variables’ distribution and help interpreting the correlations among them. These two plots must be interpreted jointly by plotting the same pairs of principal components: the scores and the loadings plots can be directly compared to point out which variables characterize the different samples and groups of samples the most.

After pre-processing the data with autoscaling (also called “unit variance scaling”) (Van den Berg et al., 2006), two different PCAs were carried out, only for the Italian varieties, by separately looking at biochemical traits and morphological features, with the aim of relating the two computed and acquired datasets with the GI values. In addition, another PCA was performed considering the morphological features calculated for both Italian and non-Italian varieties for which all morphological features could be extracted (45 out of 54).

### 2.8 Statistical analysis: considerations about the number of varieties and samples to study

The balance between the need for a broad description of the genotypes and the time needed to conduct both the GI and FESEM studies led to the choice of performing the minimum number of experimental replicates to ensure statistical representativeness of the results. A narrower selection of varieties would have allowed for performing more replicates but spanning the “genotypes space” was among our top research priorities.

For this reason, regarding the image analysis, three grains per variety were selected and scanned, taking three images at different locations of the grain. The standard deviations reported in **Tables 3** and 4 arise from these numbers of replicates.

Based on these considerations, the present study can be also viewed in an “exploratory” perspective, which was also enabled by the use of PCA, a holistic multivariate approach.

Due to the complexity of the topics covered, some methods are not explained in a sufficiently exhaustive manner to allow what the authors have done to be repeated; for this reason, readers needing more information about the materials and methods used are invited to directly contact the authors.

## 3 Results

### 3.1 Glycemic Index evaluation and biochemical analyses

GIs were calculated “*in vivo*” for each of the considered 25 Italian rice genotypes, with respect to glucose, used as standard (**Table 3**), on the basis of the glycemic curves detected in 10 healthy volunteers (**Supplementary Figures 4-6**).

**Figure 1:**
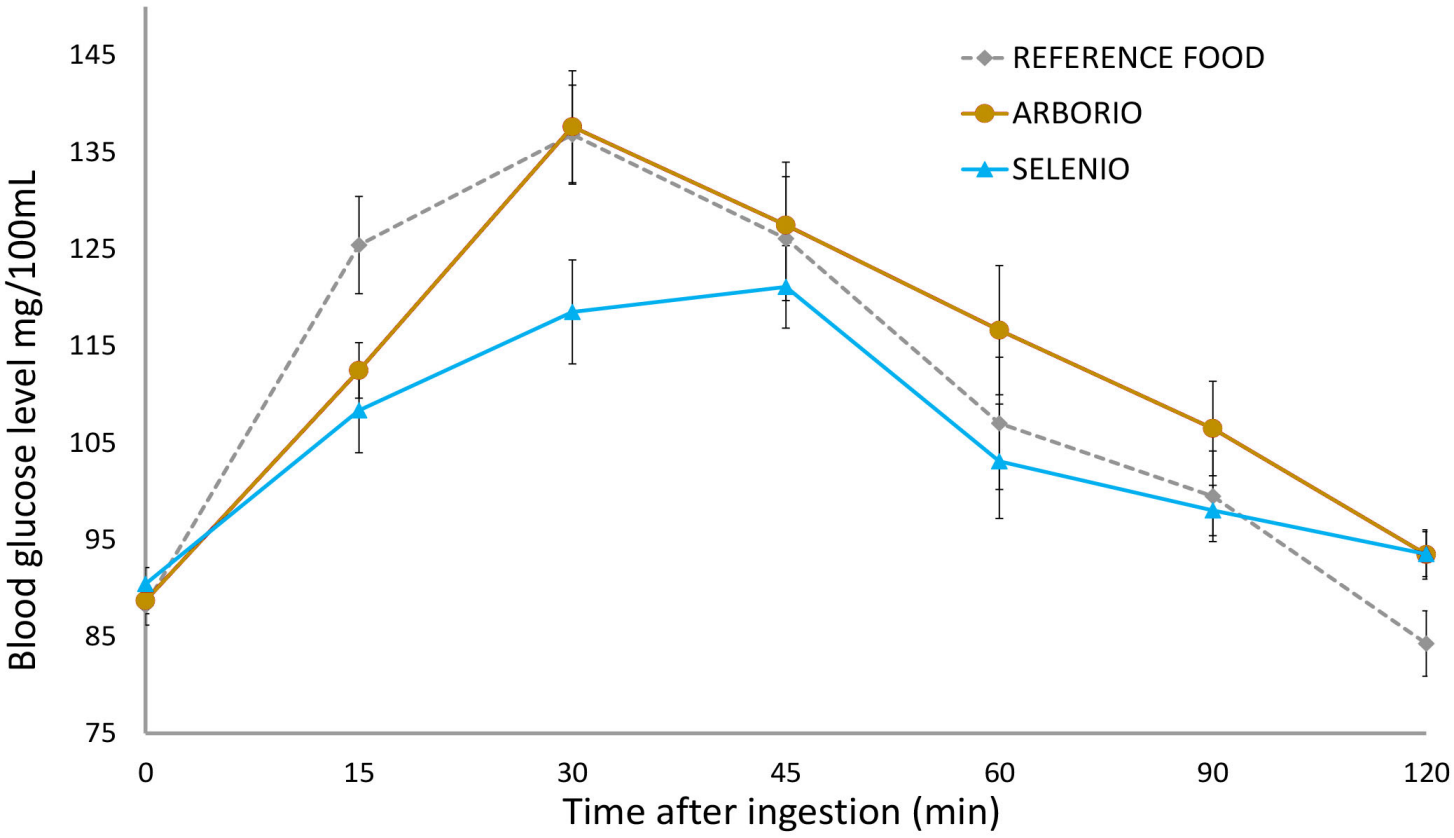
Postprandial glycemic curves, the area of which was used to calculate the Glycemic Index of test food with respect to the reference food. The graph shows the average blood glucose levels (± SD) measured after the ingestion of cooked milled rice of the varieties Selenio (GI = 49.15 ± 6.55) and Arborio (GI = 92.31 ± 8.35) with respect to the glucose solution used as standard (GI = 100).

10 genotypes showed a high GI, overcoming 70; in particular four of them (Arborio, Lince, Duilio and Castelmochi) exceeded a value of 85, with Arborio showing the highest value (92.31 ± 8.35) (**Figure 1**, **Supplementary Figure 4**). Other 10 genotypes were found to show a medium GI (between 56 and 69) (**Supplementary Figure 5**), and five genotypes showed a low GI (≤ 55); among the last, we found the cultivars Argo and Selenio, and the Enr-18215, Enr-18328 and Enr-18433 advanced lines, with Enr-18433 and Selenio showing the lowest values (49.21 ± 6.59 and 49.15 ± 6.55 respectively) (**Figure 1**, **Supplementary Figure 6**).

Biochemical analyses were also performed on the same milled rice samples (**Table 3**). Protein content (g/100 g) was found to vary from 5.64 ± 0.35, in Puma, to 7.78 ± 0.48, in Duilio.

The highest contents of total fat substances were registered in the variety Argo (also characterized by the highest average porosity and showing one of the lowest GI values), reaching 1.08 ± 0.073 g/100 g milled rice, and in the waxy variety Castelmochi, reaching 1.44 ± 0.093 g/100 g. Instead, the lowest content of total fat substances (0.57 ± 0.048 g/100 g) was registered by the Arborio variety that is also characterized by the highest GI value.

The lowest content of ashes (0.21 ± 0.04 g/100 g milled rice) was observed in the Selenio variety (showing the lowest GI), followed by Enr-18126 selected line (0.22 ± 0.04 g/100 g), Lince (0.34 ± 0.04 g/100 g) and Enr-18328 (0.35 ± 0.04); hence, three of four varieties characterized by a low ashes content, are also characterized by a low – medium GI. The highest ashes content was observed in the variety Valente (1.08 ± 0.07 g/100 g), followed by the waxy variety Castelmochi (0.66 ± 0.05 g/100 g), and by Puma (0.61 ± 0.05 g/100 g), Padano (0.6 ± 0.05 g/100 g), Duilio (0.6 ± 0.05 g/100 g), CL35 (0.6 ± 0.05 g/100 g), Iarim (0.59 ± 0.05 g/100 g) and CL71 (0.58 ± 0.05 g/100 g), all characterized by a high GI value with the only exceptions of Iarim and Valente.

Amylose content was found to be negatively correlated to GI value (r = -0.5088) in the 25 genotypes analyzed, but is in turn positively significantly correlated (r = 0.5381) to dehusked grain Length/Width ratio (an important biometric parameter to classify rice varieties in the different commercial groups).

### 3.2 Evaluating rice grain inner structure and starch granules size and morphology

Each of the 25 genotypes used for the GI evaluation, along with 11 other Italian varieties and 18 IRRI accessions (**Table 4**), were characterized by considering the internal structure of the grain using a field emission scanning electron microscope. After fine-tuning of the sample preparation procedure, 12 micrographs were acquired through SEM from three selected representative grains for each rice variety analyzed (1 at 100X and 3 at 5000X magnification for each rice grain), thus obtaining a total of 648 images (12 x 54 genotypes).

**Table 4:** Characterization of 11 Italian rice varieties and 18 IRRI accessions through FESEM imaging.

Denomination	Average starch granules size (area) (μm^2^)			Starch granules average eccentricity	Starch granules average circularity	Estimated diameter of starch granules (μm)	Average porosity (%)		
Italian rice varieties									
CL31	34.48	±	10.37	0.72	0.39	6.63	5.64	±	1.85
CL80				0.73	0.38		0.40	±	0.26
CL510	50.55	±	0.65	0.74	0.39	8.03	5.30	±	0.71
Cripto	33.24	±	2.15			6.51	6.15	±	0.59
CRW3	35.61	±	18.79	0.68	0.40	6.74	6.35	±	0.54
Dedalo	64.81	±	25.09	0.73	0.38	9.09	2.14	±	2.86
Drago	75.02	±	32.28	0.71	0.39	9.78	6.35	±	0.20
Europa	30.75	±	1.26	0.73	0.38	6.26	6.28	±	0.27
Italmochi				0.68	0.41		1.09	±	0.29
Pegaso	69.83	±	38.27	0.71	0.41	9.43	1.33	±	1.41
Prometeo	26.69	±	0.46	0.71	0.41	5.83	0.92	±	0.22
International Rice Research Institute accessions									
Kaluheenati	29.39	±	4.86	0.74	0.37	6.12	5.66	±	1.52
Mahsuri	28.22	±	7.01			6.00	5.74	±	1.10
Hetadawee	53.45	±	13.04			8.25	6.52	±	0.67
Pajam	26.70	±	0.72	0.73	0.40	5.83	5.97	±	0.24
Kahawanu				0.72	0.36		1.66	±	0.61
IR 42	54.75	±	19.47	0.74	0.40	8.35	0.69	±	0.13
IR 6	66.95	±	41.65	0.73	0.42	9.24	1.64	±	1.87
IR 50	87.63	±	9.13	0.74	0.40	10.57	6.31	±	0.34
IR 64	75.89	±	41.41	0.73	0.39	9.83	4.16	±	3.52
IR 4630-22-2-5-1-3	21.04	±	11.69	0.72	0.40	5.18	5.18	±	0.55
Cisokan				0.72	0.40		0.52	±	0.01
Taichung Sen 17	32.22	±	1.77	0.71	0.39	6.41	6.13	±	0.19
Doongara	29.00	±	2.17	0.71	0.39	6.08	6.36	±	0.05
Sinandomeng							0.99	±	0.80
Iac 165	52.87	±	29.26	0.74	0.39	8.21	4.27	±	3.14
Fedearroz 50	89.17	±	1.99	0.72	0.42	10.66	0.79	±	0.39
Cypress	84.12	±	7.16	0.71	0.39	10.35	0.89	±	0.21
Swarna	49.62	±	29.61			7.95	4.75	±	3.37

Values ± standard deviation are reported.

Starch granules diameters were estimated from their average area, considering them approximately round, finding values between 5.83 µm (in Prometeo and Pajam), and 12.53 µm (in Lince). A significant negative correlation was observed between starch granules average diameter and the ashes content (r = -0.5397).

The average percentage of endosperm porosity resulted greatly variable, showing the lowest values in CL80 (0.40 ± 0.26) and Selenio (0.40 ± 0.28), and the highest in Argo (6.72 ± 0.39). This parameter resulted inversely correlated to the average starch granule size (μm^2^) (r = -0.5822) in the 54 varieties analyzed, excluded those in which the latter resulted undeterminable. The peculiar endosperm structure of some varieties made it impossible to calculate starch granules size.

Starch granules average eccentricity and circularity were calculated. Starch granule circularity was found to be significantly positively correlated to starch granule size (r = 0.4562), showing a stronger correlation in Italian varieties (r = 0.4872). A statistical correlation was observed between starch granules average eccentricity and the content of ashes (r = 0.5181), resulting stronger (r = 0.5216) without considering Valente (characterized by the highest ashes content).

### 3.3 Endosperm physical structure in crystalline and chalky rice genotypes

**Figure 2:**
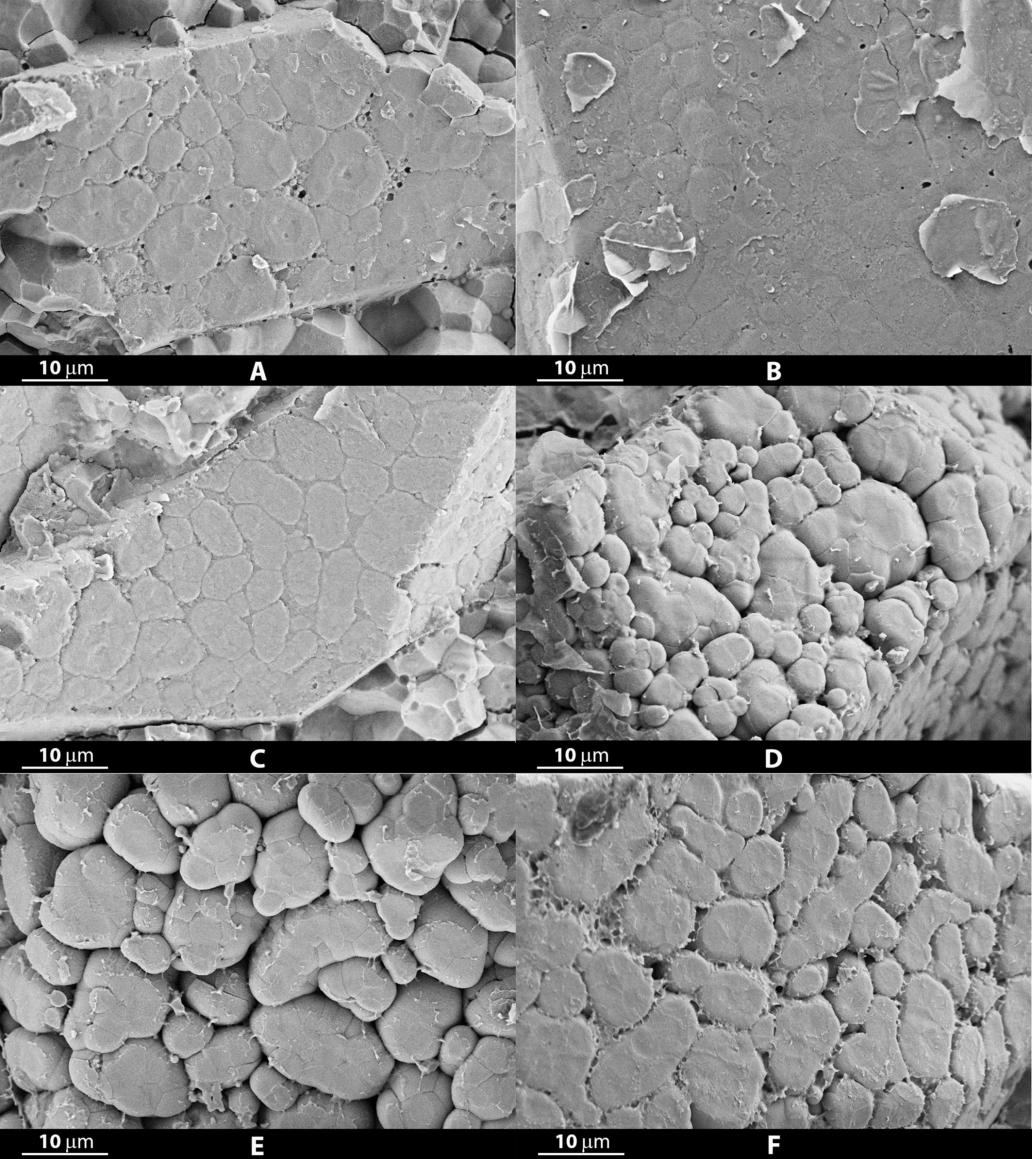
Micrographs obtained at a 5000X magnification from grains of crystalline rice varieties Selenio **(A)**, Valente **(B)** and CL80 **(C)**, and chalky rice varieties Argo **(D)**, Carnaroli **(E)** and CL388 **(F)**.

Dividing varieties characterized by a crystalline (or almost crystalline) endosperm (**Figures 2A**) from those whose average chalky fraction was estimated equal or higher than 10% (**Figures 2D**), we observed that the crystalline ones seemed to show a lower average percentage of porosity (2.65 ± 2.26) and a larger average dimension of starch granules (62.39 ± 27.33 µm^2^, corresponding to 8.7 µm in diameter). Instead, the ones having a more extent chalky fraction of the grain, showed a higher average percentage of porosity (5.15 ± 1.53) and a smaller average starch granules size (45.23 ± 15.01 µm^2^, corresponding to 7.5 µm in diameter).

No differences in average eccentricity (0.72) and average circularity (0.4) of starch granules were observed between the two groups. Nevertheless, at a visual observation of micrographs, the starch granules of the varieties with a more compact structure and with fewer empty spaces appear generally to show a more polyhedral shape, with sharp edges, while those with greater porosity present granules with a less polygonal shape.

As regards the abovementioned subdivision of the varieties between crystalline (or almost crystalline) and those with a chalky fraction greater than 10%, the average GI values resulted similar for the two groups: respectively 64.39 ± 12.48 and 68.39 ± 10.87.

Without considering waxy rice varieties, having a completely different structure, a significant positive correlation was observed between the porosity of the grains (%) and their average chalky fraction (%) (r = 0.4722), resulting higher in Italian genotypes (r = 0.5927). Furthermore, dehusked grains average width (mm) was also found to be correlated with both average porosity (r = 0.5483) and average percentage of chalky fraction (r = 0.6934) in non-waxy Italian rice varieties.

### 3.4 Varieties characterized by a peculiar endosperm structure

**Figure 3:**
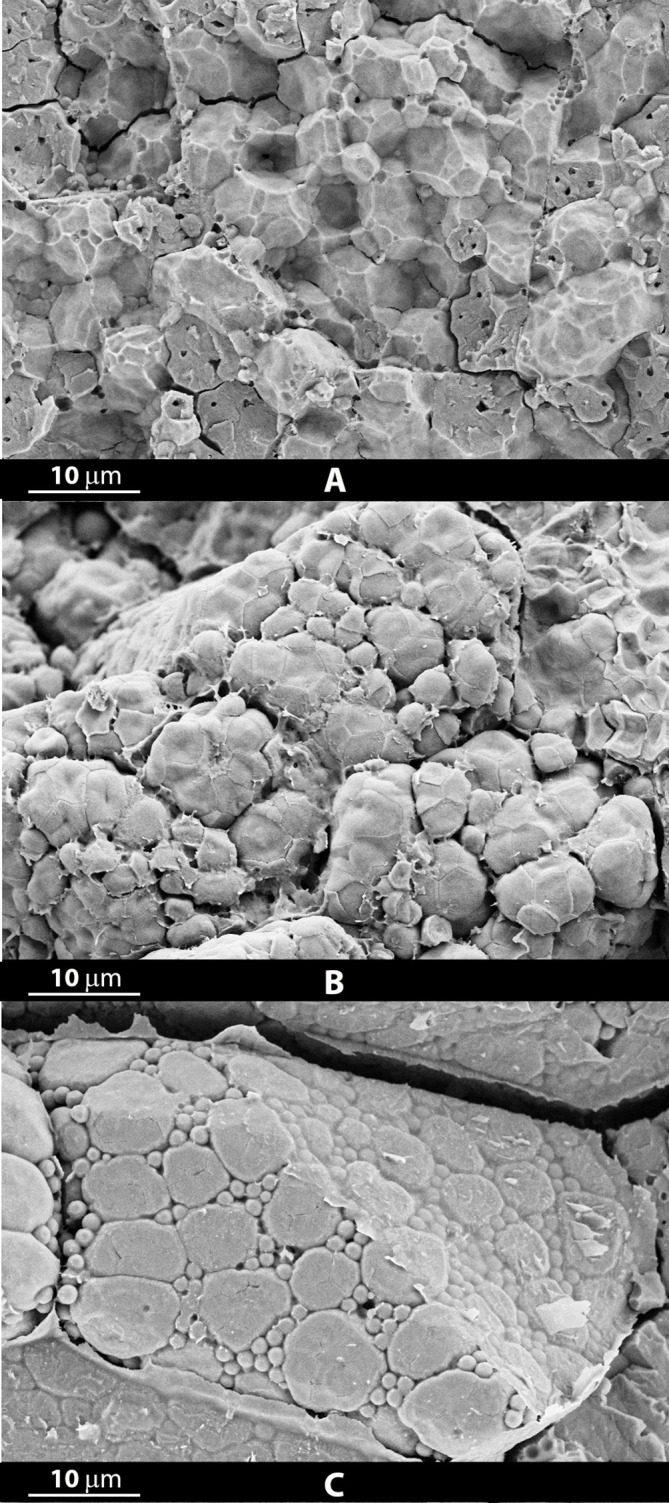
Micrographs obtained at a 5000X magnification from grains of rice varieties characterized by a peculiar inner structure, completely different from all the others observed: Italmochi **(A)**, CRW3 **(B)** and Dedalo **(C)**.

Concerning the waxy rice varieties Castelmochi, Italmochi and CRW3, we observed that both Castelmochi and Italmochi were characterized by a low average percentage of porosity (respectively 0.83 ± 0.23 and 1.09 ± 0.29), showing a peculiar endosperm structure where starch granules appeared almost indistinguishable and fused together in an uneven mass, which made it impossible to automatically determine their size (**Figure 3**). On the contrary, CRW3 showed a completely different structure, with a higher percentage of porosity, equal to 6.35 ± 0.54, comparable to that of many varieties with a chalky endosperm, and its starch granules appeared more easier distinguishable, having an average dimension of 35.61 ± 18.79 μm^2^ (6.74 μm in diameter) (**Figure 3**). Even if starch granules morphology wasn’t determined for the variety Castelmochi, starch granules of Italmochi and CRW3 showed average circularity values comparable to those observed in the non-waxy varieties (0.41 and 0.40 respectively), but the values of average eccentricity (0.68 for both) resulted the lowest observed.

A peculiar structure, clearly different from all the others, was observed in micrographs obtained from a grain of the variety Dedalo at 5000X magnification, with the presence of big roundish and smooth granules and many smaller round structures (**Figure 3**).

### 3.5 Exploratory analysis results

**Figure 4:**
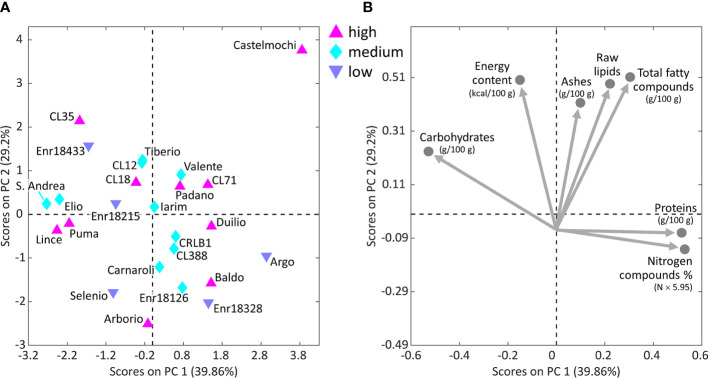
Scores plot **(A)** with the distribution of the 25 Italian genotypes analyzed by principal component analysis (PCA) on the results of the biochemical analyses performed on milled rice, colored on the basis of their high, medium or low GI, and corresponding loadings plot **(B)** with biochemical traits.

Three principal component analysis (PCA) models were built. The first model was obtained from the biochemical properties data concerning the 25 Italian varieties whose GI was evaluated (**Figure 4**), finding no relations between GI and the considered properties.

**Figure 5:**
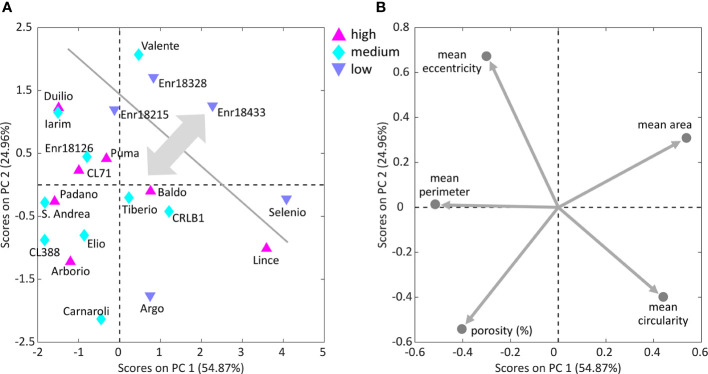
Scores plot **(A)** with the distribution of 21 of the 25 Italian genotypes analyzed by PCA on starch granules characteristics and endosperm average porosity, colored on the basis of their high, medium or low GI, and corresponding loadings plot **(B)** with starch granules features. The physical structure of 4 of the 25 varieties could not be analyzed by the *ad hoc* developed algorithm, due to the strongly prevalent presence of compact structures, thus causing some missing values in the data table.

The second PCA model was built on the internal structure features data resulting from SEM image analysis and considering the additional information about GI for 21 of the characterized Italian varieties. Castelmochi, CL12, CL18 and Enr-18433 were not included in this PCA model due to the impossibility of automatically extracting features related to the average size of starch granules. The evaluation of scores plot (**Figure 5**) does not highlight the presence of clusters among the samples, but from the loadings plot (**Figure 5**) some interesting trend in the variables disposition could be noticed. Two anti-correlations seem to exist: between eccentricity and circularity and between mean starch granules area and porosity.

**Figure 6:**
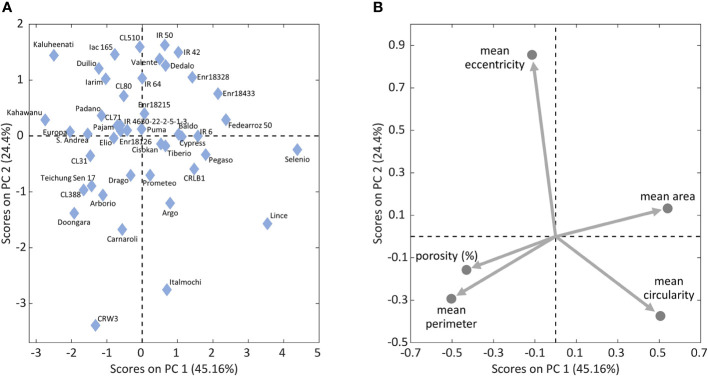
Scores plot **(A)** with the distribution of 45 of the 54 Italian varieties and IRRI accessions analyzed by PCA on starch granules characteristics and endosperm average porosity, and the corresponding loadings plot **(B)** with starch granules features. The physical structure of nine of the 54 varieties could not be analyzed by the *ad hoc* developed algorithm, due to the strongly prevalent presence of compact structures, thus causing some missing values in the data table.

A third PCA was made considering 45 of 54 Italian rice varieties and IRRI accessions, and the morphological features of their internal structure. Nine varieties were excluded because of the impossibility of extracting the average starch granules size value. By looking at the scores and loadings plots (**Figure 6**), eccentricity and circularity result as anti-correlated the samples in the scores plot are distributed along this direction in relation to how round their starch granules are. Varieties like IAC165, Duilio and Iarim show lower circularity and higher eccentricity, while varieties like Lince and CRLB1 show higher circularity values (round shaped grains).

From the same PCA results, by focusing again on the loadings plot, also the anti-correlation between porosity and average starch granules area could be spotted again.

## 4 Discussion

### 4.1 Glycemic Index evaluation in 25 Italian rice varieties

Several studies have calculated the GI of rice varieties using different methods, both “*in vivo*”, i.e., on the basis of the glycemic curves detected in volunteers subjected to tests, and “*in vitro*” (simulating digestion) (Miller et al., 1992; Hettiarachchi et al., 2001; Indrasari et al., 2010; Fitzgerald et al., 2011; Oko et al., 2012; Nisanka and Ekanayake, 2016; Kumar et al., 2018; Bhagya, 2019; Dwiningsih and Alkahtani, 2023; Srikaeo, 2023). However, currently available data are still limited compared to the thousands of rice varieties grown all over the world, especially as regards varieties cultivated in Italian and European rice area. Implementing knowledge on the GI of commercially available temperate Japonica rice varieties is essential to provide clear information to consumers, thus protecting their health.

Exceeding our expectations, a great variability in GI values was observed among the studied Italian genotypes (**Figure 1**; **Table 3**). 5 of them were found to have a low GI, <55 (Selenio, Argo, Enr-18215, Enr-18328, Enr-18433) and 10 varieties showed a medium GI value, between 56 and 69 (Carnaroli, CL12, CL388, CRLB1, Elio, Enr-18126, Iarim, S. Andrea, Tiberio and Valente). Instead, the varieties Arborio, Lince, Duilio, Castelmochi, Padano, Puma, Baldo, CL18, CL35, CL71 were found to have a high GI (>70). Unexpectedly, the highest GI value was registered for Arborio (92.31 ± 8.35) (having a Long A chalky grain), followed, with similar values, by Lince (88.93 ± 9.22) and Duilio (86.22 ± 10.18), both characterized by an almost crystalline endosperm, and by the Castelmochi variety (84.71 ± 10.65) that, having a glutinous endosperm (and a particularly low amylose content), was supposed to show the highest GI, according to the results of previous publications (Srikaeo, 2023).

The Carnaroli cultivar, often considered the best Italian variety to prepare a good “risotto” dish, was found to have a 64.17 ± 6.50 GI value, in agreement with the value previously reported (64 ± 11) referring to a commercial sample of rice belonging to the “Carnaroli” group (Scazzina et al., 2016).

GI values calculated in our study vary between 49 and 92, the same range reported by Fitzgerald et al., analyzing a wider number of genotypes (Fitzgerald et al., 2011). This bears witness to the richness of Italian rice cultivation which has drawn for centuries from different sources, introducing dozens of varieties from foreign countries and later crossing them with each other, thus creating a wide genetic variability.

According to our results, 2 rice varieties and 3 selected lines among the 25 Japonica Italian rice genotypes analyzed are therefore suitable for feeding diabetic subjects and subjects with impaired fasting glycemia, thanks to their low GI. This represents an excellent and unexpected result, and the fact that 20% of the varieties analyzed were found to show low GI, bodes well that among the over 250 rice varieties in the Italian National Register, there may be many others with a low GI. These results show for the first time that, even among the European temperate Japonica varieties, there are some with low GI and that this characteristic is not strictly related to a very high amylose content. In addition, a plus is given by the fact that the round grain variety Selenio, showing the lowest GI, has always been among the most cultivated varieties in Italy in recent years, despite its release dates back to 1987.

Notwithstanding the recent growing interest for low GI rice, analyzing dozens of varieties from different countries, Fitzgerald et al. highlighted how the aspects related to the glycemic response of rice have been neglected by the breeding programs of the last few decades, which have unintentionally led to an increase in GI (Fitzgerald et al., 2011). Based on our results, this consideration does not seem to apply to Italian varieties, as among those with a low and medium GI there are both old and recent ones, but the number of genotypes analyzed (25) compared to those on the market (over 250), is too small to draw conclusions.

### 4.2 Investigating the role of amylose and other factors in determining Glycemic Index

Total amylose content is usually considered more important than its structure in determining GI: rice varieties with higher amylose content require a higher gelatinization temperature due to restrained swelling by amylose, hence a longer cooking time, compared to the other ones (Fitzgerald et al., 2011); furthermore, the formation of complexes between amylose and lipids during heating reduces enzymes access to starch (Goddard et al., 1984) and the presence of a high amylose content increases starch retrogradation during cooked rice cooling (Chung et al., 2006; Ranawana et al., 2009), reducing its digestibility due to the formation of resistant starch (RS) (Boers et al., 2015; Ngo et al., 2023). A higher RS content is considered to correspond to a slower digestion and a lower GI, with a significant negative correlation between the two parameters (Kumar et al., 2018).

Fitzgerald et al. (2011) reported a reverse correlation between amylose content and predicted GI (r = -0.8544) (Fitzgerald et al., 2011); in the characterized Italian rice varieties the correlation between amylose and GI resulted significant too (R = -0.5088), but not as strong as reported by other authors. Hence, although at most of the low-GI varieties known in literature, corresponds a high amylose content (Miller et al., 1992; Fitzgerald et al., 2011), our results suggest that this is not necessarily a discriminating trait, since the Selenio variety, for example, showed a low GI value despite having a medium-low amylose content, thus signifying that many other factors could be involved in determining GI. According to Miller et al. (1992), indeed, only large differences in amylose content, leading to higher RS content after cooked rice cooling, cause relevant effects on rice GI (Miller et al., 1992).

Our results agree with the fact that low-amylose Japonica rice genotypes were reported to show a slower and reduced degree of digestion compared to low-amylose Indica, while high-amylose Japonica rice varieties show a faster and higher degree of digestion compared to high-amylose Indica (due to the presence, in high-amylose Japonica varieties, of more rapidly digestible starch and less slowly digestible starch and RS) (Hu et al., 2004; Boers et al., 2015). Thus, between high and low amylose Japonica rice varieties, the differences in digestion rate are smaller and the role of other factors may increase. Juliano pointed out the role of varietal differences in gelatinization temperature and degree of branching of starch fractions, in determining nutritional properties such as RS and GI, in addition to the amylose content (Juliano, 1992).

Other factors could also be involved in GI variations: for example, Indica non-waxy starch has been reported to contain about three times more protein bodies than Japonica rice starch having a similar amylose content (Villareal and Juliano, 1989), and knowing that starch-protein complexes have a role in starch digestion and in resistant starch formation (Ngo et al., 2023), this could partially explain the differences observed among Japonica rice varieties, where the correlation between GI and amylose content is not so evident as in Indica varieties. Furthermore, long-chain amylopectin was observed in cultivars with high amount of slowly digestible starch, indicating that, in addition to amylose content, differences in amylopectin fine structure also affects starch digestion properties (Benmoussa et al., 2007).

### 4.3 Use of scanning electron microscopy to evaluate morphological traits of the endosperm structure in rice

Starch, representing the main chemical storage form of energy in plants tissues, is synthesized in the amyloplast, a differentiated plastid where carbon, translocated from source tissues, is converted and stored. In rice endosperm each amyloplast produces compound granules consisting of several small sharp-edged granules close together (Watson and Dikeman, 1977; Yun and Kawagoe, 2010), whose typical polyhedral shape is probably due to their compression during development and filling (Juliano, 1972); whereas, in other cereals, each amyloplast generates a single bigger starch granule (Yun and Kawagoe, 2010).

Starch granules are composed by semi crystalline and amorphous regions creating concentric growth rings (Vandeputte and Delcour, 2004; Fujita, 2014; Patindol et al., 2015). Those with higher amylose content are supposed to have more extensive hydrogen bonding, able to confer them a more crystalline structure than those with a low amylose content (Panlasigui et al., 1991; Lopez-Rubio et al., 2008).

Only observing starch granules directly from their botanical source, it is possible to evaluate their actual arrangement and aggregation inside the rice grain, to understand how the physical structure of the grain can affect characteristics such as its abilities to stretch and to absorb water upon cooking and its organoleptic characteristics, as well as, putatively, other features such as GI.

The study of the internal structure of the grain, and in particular of starch granules morphology, dimensions, and of the spaces among them, was carried out by observing longitudinal sections of the grains through FESEM imaging. Micrographs were obtained at the same magnification level (5000X) so that relative comparison of each variety can be made. The observation of the grain structure gave us information concerning the existing variability among genotypes in starch granules morphology and distribution, as well as in endosperm average porosity (**Tables 3** and **4**). Hence, this study adds another brick to the knowledge of rice grain microstructure by assessing the existence of amazing differences in morphological features among different rice genotypes.

Comparing micrographs obtained from different samples, at least in non-waxy varieties, it is possible to observe compound starch granules, appearing as rounded structures, made up of “smaller wedges” (the individual starch granules), looking similar to soccer balls. Small starch granules, composing the amyloplast, are separated by a septum-like structure, or cross-wall (inner envelope membrane) and each compound starch granule is enveloped by an outer membrane, at whose synthesis contribute plastid division proteins, having a critical role also in amyloplast division (Yun and Kawagoe, 2010). These membranes were found to be visible by FESEM in some of the micrographs obtained at a 5000X magnification (**Figure 2**). In some cases, these membranes were more evident, perhaps due to their greater thickness, creating a sort of film over the starch granules which made it difficult to distinguish them, especially in varieties having a more compact structure, compared to other ones.

In some images, compound starch granules were grouped to form larger and elongated polygonal structures, which resemble crystals, identifiable as single cells (**Figure 2**). Rice endosperm structure has been described as composed by thin-walled cells, which are usually elongated radially on cross-sectional view, and smaller, polygonal or slightly elongated, on lateral sides of the grain, filled with compound starch granules and protein bodies (Juliano, 1992; Patindol et al., 2015). Starch granules in the peripheral cells of the endosperm are reported to be smaller and aggregated in tiny clusters separated by a proteinaceous material, compared to those in the central portion, which are more closely packed in each cell (Watson and Dikeman, 1977).

### 4.4 Determination of starch granules morphological parameters and other traits characterizing the inner structure in 54 rice varieties belonging to different product groups

The average diameter of starch granules, estimated from their average size (area) considering them approximately as round, resulted 8.15 ± 1.78 µm, in agreement with measurements made by Jane et al. (1994) on purified rice starch, who found polygonal shaped granules with diameters of 3–8 µm, much smaller than those from maize (5–20 µm) and other cereal sources (Jane et al., 1994). The extraction of morphological parameters of starch granules resulted impossible for some genotypes, for which images segmentation was not reliable: nine rice varieties showed a so compact structure that the starch granules were indistinguishable, making impossible to calculate their average size, but it was still possible to obtain porosity values (very low) for these varieties.

Starch granules size and rice grain average porosity resulted inversely correlated (r = -0.5822). Starch granules circularity was found to be correlated to their average size, showing a stronger correlation in Italian varieties (r = 0.4872), compared to the other investigated rice varieties.

A trend related to the GI can be observed in **Figure 5**, as most of the samples with low GI (namely Enr-18215, Enr-18328, Enr-18433 and Selenio) are in the top-right part of the plot. By inspecting the PCA loadings (**Figure 5**), it can be seen that these low-GI samples are characterized by high mean area, i.e., they tend to have larger starch granules, while at the same time they exhibit low porosity. On the contrary, the medium- and high-GI samples are located diagonally in the opposite direction, thus having higher porosity and lower mean area, i.e., generally smaller starch granules. Selenio, the variety with the lowest GI, was found to show one of the lowest porosity values (0.40 ± 0.28), coherently with its position, which is opposite to the direction of the porosity loading (**Figure 5**). The Argo variety, also characterized by a low GI, showed instead the highest porosity value 6.72 ± 0.39, and it is the only low-GI sample not following the detected trend. However, this is not unexpected because GI is indeed a multiparametric value that could be related to other chemical, biological and/or morphological aspects.

We can broadly distinguish the characterized Italian varieties in three main groups, based on their average porosity: a group of varieties characterized by a very low porosity value (<1.7%), including mainly those with a crystalline endosperm (**Figures 2A–C**); a second group characterized by a high porosity value (> 5%) including the most of Long A varieties for the National market among those considered (e.g., Arborio, Carnaroli, Padano, S.Andrea and the more recently released variety CL388) (**Figures 2D–F**); and a third group with intermediate porosity values between 2.1 and 4.2% including the widely appreciated Baldo variety and other more recent varieties with intermediate characteristics. Porosity resulted correlated with the extension of the chalky fraction of the endosperm and with grain width in Italian genotypes, thus confirming being a main characteristic of Long A grain varieties for risotto cooking.

The structure of the chalky area of the endosperm, typical characteristic of many Italian Short and Long A grain varieties for national consumption, was generally more regular and better defined, with well separated starch granules, thus resulting more adapt to an automatic processing of the images, if compared to not chalky areas (**Figures 2D**; **Supplementary Figure 3**). The crystalline part of the endosperm, which in some cases represents the entire grain section, appears morphologically completely different, with starch granules joined together into larger structures (**Figures 2A**).

It is known that starch granules development begins from the innermost cells of the endosperm, spreading to the outer cells centrifugally, hence, the peripheral cells, situated near the aleurone layer, are the last to be filled during grain development, about one month later the beginning of the process (Hoshikawa, 1993; Matsushima et al., 2010). Matsushima et al. suggested that the molecular mechanisms responsible for starch granules formation could change during rice caryopsis filling, giving rise to differences in starch granule morphology and distribution between the inner and outer parts of the grain (Matsushima et al., 2010). Even if those mechanisms are still unknow, this could explain the formation of the chalky part of the endosperm, which characterizes many of the Italian rice varieties, whose structure is morphologically different from that of the surrounding more crystalline part, as highlighted by micrographs, also at 100X magnification.

Italian rice varieties, and in particular those intended for the preparation of risotto, are characterized by the ability to absorb seasonings and release starch during cooking; in this way, grains blend together conferring to the dish a creamy consistency. Our results suggest that this specific characteristic could be linked to a less compact internal structure of the grains, being characterized by a higher average endosperm porosity with more distant starch granules having a smoother surface, particularly in the chalky portion of the grain, compared to varieties characterized by a crystalline endosperm, such as those most appreciated in northern-European countries, which show a compact structure with larger starch granules and a reduced average porosity.

The amyloplasts packing variation, caused by genetic or environmental factors, is thought to determine milled rice translucent, opaque or chalky appearance (Patindol et al., 2015), so the percentage of porosity can be considered a measure of this parameter. According to literature, chalky parts of rice grains, whose formation is favored by high temperatures, looks white because they are not completely filled with starch granules, having a less defined polyhedral shape and small gaps that cause light scattering (Hoshikawa, 1993). We can suppose that, although chalkiness is actually different from milky grains, even the chalky part of the grain of Italian rice varieties appears less translucent than the crystalline portion, precisely because of existing morphological differences, above all, in the arrangement of starch granules and endosperm porosity, causing a different light refraction. A linear correlation (r = 0.593) was indeed observed between average porosity (%) and average chalky fraction (%) of the grain volume, in the 36 Italian genotypes analyzed, excluding the waxy ones; instead, no correlation was observed between the two parameters considering IRRI accessions, having a completely different endosperm structure, suggesting that other factors are involved.

### 4.5 Comparison of the morphological traits of the inner structure of the grain in rice varieties having an entirely crystalline, partially chalky, or glutinous endosperm

No differences were observed in the average eccentricity of the starch granules (0.72), nor in the mean circularity (0.4) between crystalline (or almost crystalline) varieties and those with a larger chalky fraction. The average amylose content, as well as GI values, resulted similar for the two groups, indicating that these differences in grain inner structure are not primarily due to the total amylose content. In rice amylose extender mutants (characterized by a particularly high amylose content, up to 40%), starch granules have been reported to be small, round and loosely packed (Yano et al., 1985); these characteristics are similar to those we observed in the chalky fraction of the grain of Italian rice varieties. We can therefore surmise that, despite the overall content of the grain, the chalky fraction of the endosperm could contain a higher amount of amylose than the outer part. If so, amylose distribution within the grain (together with the starch nanostructure), could be more effective in determining the physical structure of rice endosperm than amylose content as a whole.

Glutinous (waxy) varieties are characterized by a very low amylose content. Their inner structure appears compact, with hardly distinguishable starch granules, often fused together in clusters, resulting more uneven and irregular than in crystalline varieties. In agreement with our results, Jane et al. observed that starch granules of waxy rice are more irregular in shape and appear compound or fused (Jane et al., 1994); a similar behavior was observed also in waxy maize, where starch granules showed a modified polygonal shape with not well distinguished individual faces compared to wild type (Jane et al., 1994).

A high porosity was expected to explain the completely white appearance of the milled grains of waxy rice varieties, nevertheless, endosperm porosity was found to be greatly variable in waxy varieties. Micrographs showed a similar inner structure for the Castelmochi and Italmochi varieties, with low porosity values (0.89 ± 0.23 and 1.09 ± 0.29, respectively), comparable to that of crystalline varieties such as CRLB1 (0.83 ± 0.16); while the waxy CRW3 variety showed a totally different morphological structure with a high porosity value (6.35 ± 0.54), comparable to that of chalky rice varieties. These differences could depend on the different genetic background of these varieties, which probably affects other factors involved in starch granules morphology and distribution. Hence, according to our results, a high percentage of porosity is not a characteristic trait of waxy rice varieties. The irregular and confused structure of waxy starch granules, whose contours do not appear clearly distinct by the enveloping membranes as in non-waxy varieties, allowed us to measure their average size only in CRW3, thanks to a greater distance among granules, showing no differences compared to non-glutinous rice varieties, thus confirming, as previously reported in literature, that amylose content does not directly determine starch granules size (Matsushima, 2015).

Starch granules average circularity in Italmochi and CRW3, resulted comparable to that observed in the non-waxy varieties (0.41 and 0.40, respectively), but the values of average eccentricity (0.68 for both) resulted the lowest observed at all; this could be related to the particularly low amylose content of glutinous varieties, even if the two parameters didn’t result correlated. The waxy Castelmochi variety was also found to be characterized by the highest total fat substances among those analyzed, reaching 1.44 ± 0.093 g/100 g.

Waxy rice starch granules are reported to be characterized by a lower density than those of non-waxy rice varieties (Patindol et al., 2015). This observation, together with our results, suggests that the main structural differences between waxy and non-waxy rice varieties, conferring also their typical white appearance, could be due to their nanostructure and chemical composition (lacking of amylose) and only in lesser extent to their microstructure (starch granules morphological features and endosperm porosity).

Small round structures were observed among the compound starch granules in several images obtained at 5000X magnification, being more evident in micrographs of some varieties such as Dedalo, Swarna and Doongara, than in others. Those structures (about 2–3 µm in diameter) could correspond to smaller starch granules originated from amyloplasts division (Yun and Kawagoe, 2009), and their presence could be due to the incomplete maturation of the grains, as observed in immature waxy maize seeds (Jane et al., 1994). Nevertheless, the existence of a bimodal granule size distribution, reported in other cereal species, has never been observed in rice mature grains (Lindeboom et al., 2004). Probably, at least some of these small round structures are protein bodies (Watson and Dikeman, 1977; Juliano, 1993), whose dimensions reported in literature are between 0.5–4 µm (spherical) and 2–3.5 µm (crystalline) (Patindol et al., 2015). If so, the reason for the presence of such a high amount of protein bodies in micrographs obtained from Dedalo grains (**Figure 3**) remains unknow and could depend on the points of the endosperm in which micrographs were taken, as it is known that protein bodies distribution is not uniform throughout the endosperm, being more abundant in the outer part than in the middle (Patindol et al., 2015). Furthermore, the separation between the individual starch granules within the amyloplasts in micrographs obtained from the Dedalo variety is poorly visible, so that they appear almost as single big granules.

### 4.6 Correlations and trends among grain biochemical and biometrical traits and morphological features of the endosperm

Considering grains biometrical traits in the 25 Italian genotypes analyzed, a significative linear correlation (r = 0.5381) was observed between the amylose content and dehusked grain length/width ratio, representing an important biometric parameter to classify rice varieties in the different market groups. This means that longer and thinner grains tend to be characterized by a higher amylose content.

In this study, several compositional and structural features of rice grain were considered, leading us to observe that at a desired low GI or a higher amylose content does not correspond a specific endosperm structure in temperate Japonica rice varieties. Nevertheless, biochemical analysis pointed out some peculiar traits characterizing varieties showing a particularly high or low GI, or characterized by specific features in endosperm structure. There appears to be a weak and not significantly reverse correlation between the total content of fat substances (g/100 g) and GI in non-waxy varieties (r = -0.3265): among non-waxy rice varieties, Argo (also characterized by the highest average porosity and by one of the lowest GI values), showed the highest content of total fat substances (1.08 ± 0.073 g/100 g milled rice). Instead, the lowest content of total fat substances (0.57 ± 0.048 g/100 g) was registered by the Arborio variety that is also characterized by the highest GI value.

Surprisingly, ashes content seems to be related to different endosperm morphological traits and in lesser extent to GI: several varieties characterized by a low to medium GI were reported to show also a particularly low ashes content, with Selenio showing 0.21 ± 0.04 g ashes/100 g milled rice; on the contrary the most of the varieties showing a high ashes content were characterized by a high GI value with the only exceptions of Iarim and Valente. Hence, excluding Valente (having the highest ashes content), this parameter appears to be weakly directly related to GI (r = 0.3259). Furthermore, ashes content resulted to be significantly correlated to starch granules average eccentricity (r = 0.5181) and inversely correlated to their estimated average diameter (r = -0.5397). Hence, a putative role of ashes content with respect to other parameters, including GI, emerges, which could be explained by differences in starch granules density and nanostructure, such as those in chains length and arrangement of amylose and amylopectin molecules, or in the composition of the amyloplast membranes that could affect the mineral content.

Although the GI values of the 18 IRRI accessions considered in this study are reported in literature, we chose to avoid taking them into consideration with respect to other evaluated parameters, as these values were calculated with different methodologies, both *in vivo* and *in vitro*; using glucose as a reference food in some cases and white bread in others. Considering that even the conversion factor from a GI value referring to white bread to one referring to glucose is not precisely defined and varies slightly in different publications. Therefore, the values reported in the literature can give us a general indication regarding whether the GI is high or low but they are in any case poorly comparable with each other and with those obtained by us. Because of this, to avoid confusion, although the GI values reported in other publications were available, they were not considered and reported in this paper.

### 4.7 A specific inner structure of the grain appears to be a peculiar characteristic of each variety of rice

Even though genotype plays a major role in determining starch properties and structure, environmental factors are also able to influence starch qualities, protein and lipids synthesis, as well as starch granules size distribution (Kongseree and Juliano, 1972; Patindol et al., 2015); particular growing condition can also impact on the amylose content, that increases in excess of nutrients of zinc and potassium as well as in flooding conditions, and decreases in salinity conditions. (Dwiningsih and Alkahtani, 2023). Hence, the same variety harvested in different seasons and in different locations cannot guarantee the same quality, thus making particularly difficult to precisely define the characteristics of each variety and to conduct breeding programs aimed to improve rice grain quality. Efforts should be made to identify genotypes characterized by more stable quality traits and to test selected lines in different cultivation areas to better assess their features. Despite this, our results showed a common endosperm structure for similar varieties, with great differences among varieties belonging to different market groups or destined to different purposes, suggesting that the internal structure, albeit with a certain variability among grains, can be considered characteristic of each variety, or at least of a variety group, representing a sort of fingerprint.

## 5 Conclusions

This study highlights the existence of a great variability among the rice genotypes analyzed, determining for the first time the GI and investigating the internal structure of the grain in a large set of Italian varieties. Five of them showed a particularly low GI, thus resulting already suitable for people affected by diabetes or other metabolic disorders. In the studied genotypes, the correlation between amylose and GI (r = -0.5088) was not found to be so strong as previously reported by other authors, indicating that many other compositional and structural factors are involved. Hence, amylose content does not seem to be decisive in determining the GI of temperate Japonica rice varieties.

From the point of view of the evaluation of the endosperm inner structure, with particular reference to starch granules size and arrangement, it emerges that crystalline varieties are characterized by low porosity, having bigger and more tightly packed compound starch granules, compared to chalky short-grain and Long A grain varieties destined to the Italian national market (risotto), which are characterized by a higher average porosity of the grain. Glutinous varieties, on the other hand, are not characterized by a defined level of porosity.

The use of a multivariate exploratory approach based on PCA also allowed inspecting the relationships among the morphological parameters algorithmically extracted from the SEM images: an interesting trend related to the GI could be observed, showing that most of the samples with low GI tend to have larger starch granules, while at the same time they exhibit lower porosity values. On the contrary, the medium- and high-GI samples showed the opposite features (i.e., higher porosity and lower mean area).

All information obtained in the study will be useful for breeding programs to develop varieties with the best characteristics according to their intended use, focusing on grain quality to fully satisfy consumers. Furthermore, the low GI genotypes already identified, will represent the starting point for carrying out specific crossbreeding programs to develop new low GI rice lines, thus meeting the needs of Italian and European diabetic consumers.

## Data availability statement

The original contributions presented in the study are included in the article/**Supplementary Material**. Further inquiries can be directed to the corresponding author.

## Ethics statement

The studies involving humans were approved by local ethics committee ethical code number: 2608/11012022. The studies were conducted in accordance with the local legislation and institutional requirements. The participants provided their written informed consent to participate in this study.

## Author contributions

FH: Conceptualization, Project administration, Writing – review & editing. FS: Conceptualization, Supervision, Validation, Writing – review & editing. MR: Conceptualization, Supervision, Writing – review & editing. EC: Visualization, Writing – original draft. LC: Resources, Writing – review & editing. EM: Resources, Writing – review & editing. CS: Writing – review & editing. GG: Investigation, Writing – review & editing. AChia: Investigation, Methodology, Writing – review & editing. MS: Data curation, Formal Analysis, Methodology, Software, Validation, Visualization, Writing – review & editing. NC: Data curation, Formal Analysis, Methodology, Software, Validation, Visualization, Writing – review & editing. AChio: Methodology, Writing – review & editing. CG: Investigation, Writing – review & editing. GB: Investigation, Writing – review & editing. ACav: Investigation, Writing – review & editing. FM: Investigation, Writing – review & editing. GM: Investigation, Writing – review & editing. AM: Investigation, Writing – review & editing. ZP: Investigation, Writing – review & editing. MP: Investigation, Writing – review & editing. AT: Investigation, Writing – review & editing. DG: Formal Analysis, Writing – review & editing. SP: Formal Analysis, Writing – review & editing. RM: Supervision, Writing – review & editing.
