# Supplementary material for: Endosperm structure and Glycemic Index of Japonica Italian rice varieties
Source: Front Plant Sci. 2024 Jan 5;14:1303771. doi: 10.3389/fpls.2023.1303771 (PMC10796725; doi:10.3389/fpls.2023.1303771)
Supplement: Supplementary file 1 [file DataSheet_1.docx]

Supplementary Material

**Endosperm structure and Glycemic Index of Japonica Italian rice varieties**

**Filip Haxhari; Francesco Savorani, Mariangela Rondanell, Enrico Cantaluppi*, Luigi Campanini, Edoardo Magnani, Cinzia Simonelli, Gentian Gavoci, Alessandro Chiadò, Mattia Sozzi, Nicola Cavallini, Angelica Chiodoni, Clara Gasparri, Gaetan Claude Barrile, Alessandro Cavioni, Francesca Mansueto, Giuseppe Mazzola, Alessia Moroni, Zaira Patelli, Martina Pirola, Alice Tartara, Davide Guido, Simone Perna, Roberto Magnaghi.**

*** Correspondence:** Enrico Cantaluppi: e.cantaluppi@enterisi.it

# Supplementary Figures and Tables

## Supplementary Figures


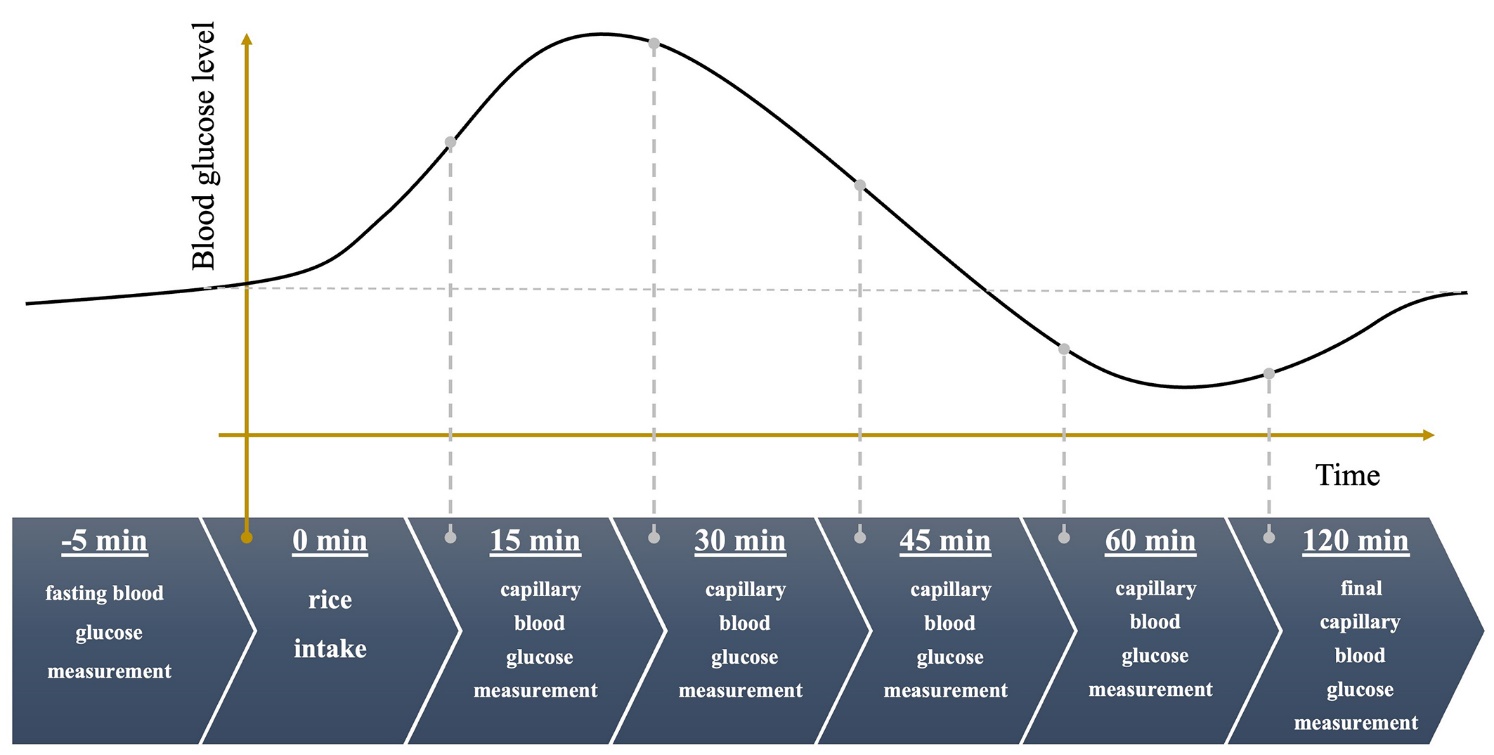


**Supplementary Figure 1.** A diagram summarizing the in vivo study.


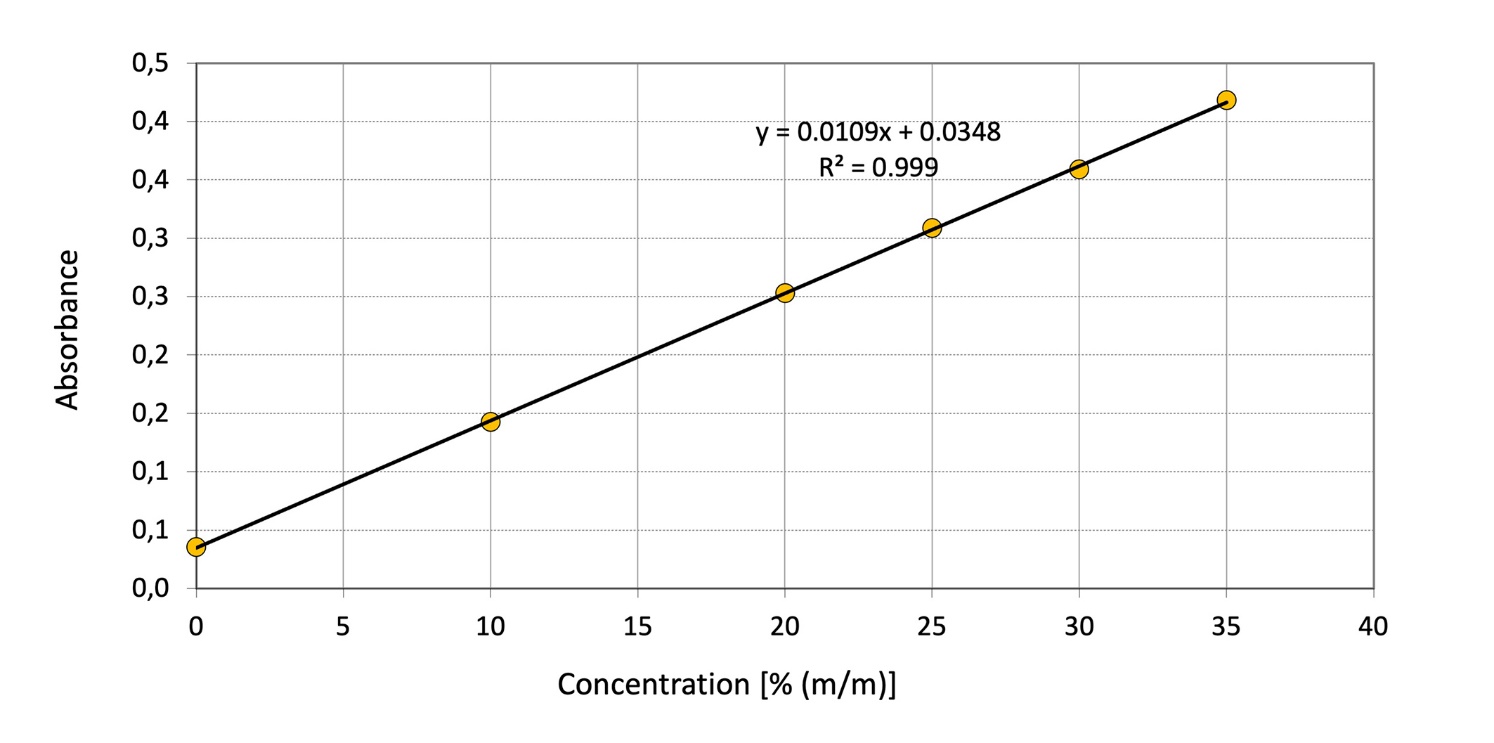


**Supplementary Figure 2.** Calibration curve for apparent amylose content quantification in accordance to ISO 6647-1:2020 determined with the spectrophotometer (set of calibration solutions: amylose mass fraction in milled rice of 0, 10, 20, 25, 30, 35%; correlation coefficient: 0.999).


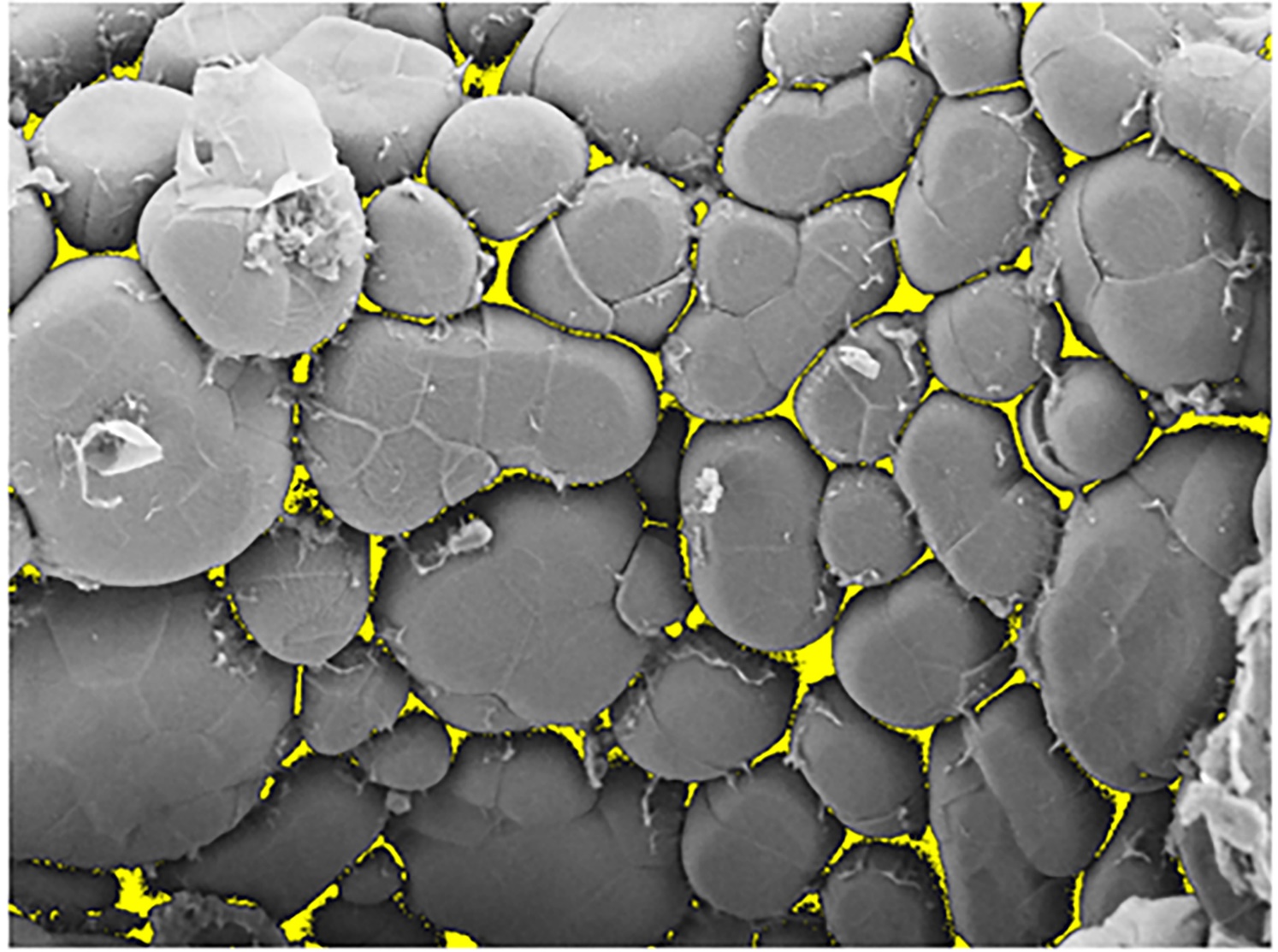


**Supplementary Figure 3.** The image shows graphically how endosperm porosity has been evaluated. The ad hoc algorithm developed for image analysis and processing, using the MATLAB software development environment, allowed the recognition of the areas represented by starch granules and amyloplasts (grey) and the gaps among them (yellow), distinguishing empty spaces from shadows. The average percentage of porosity of the endosperm was thus automatically calculated as well as the average size of the starch granules (μm^2^).


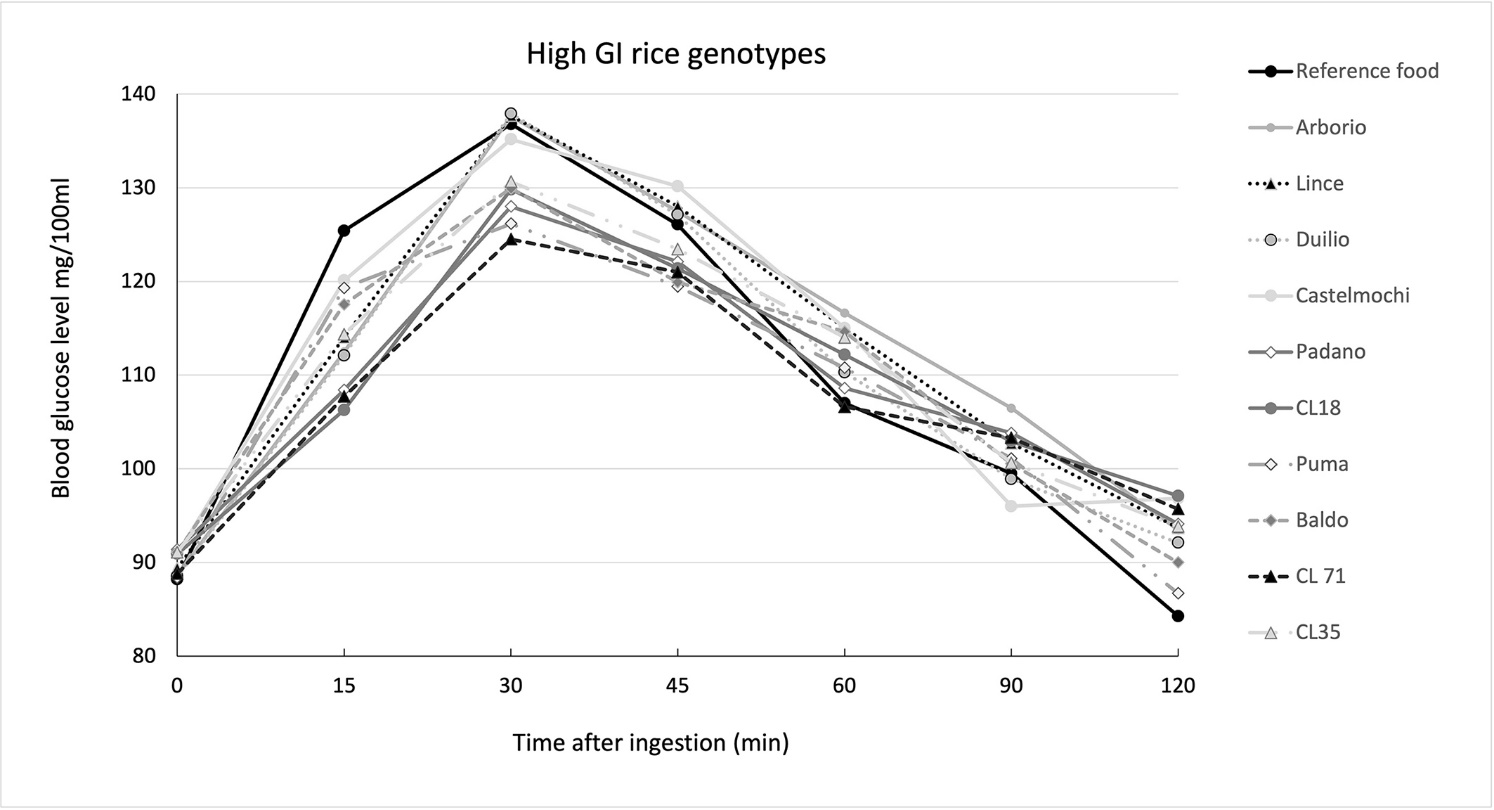


**Supplementary Figure 4.** Postprandial glycemic curves of ten Italian rice genotypes showing a high Glycemic Index (> 70). The graph shows the average blood glucose levels measured in ten healthy volunteers after the ingestion of cooked milled rice, with respect to the glucose solution used as standard (GI = 100).


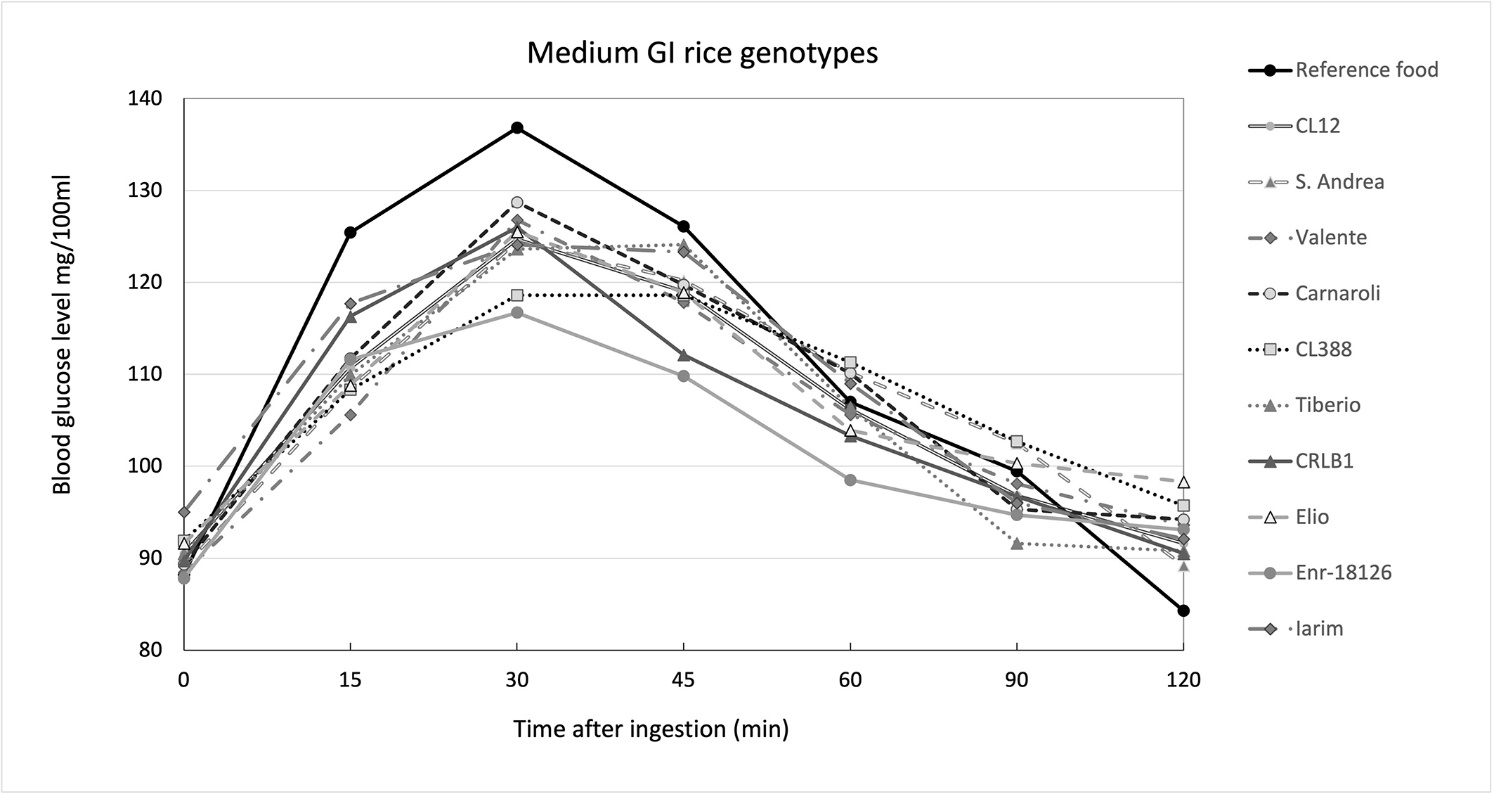


**Supplementary Figure 5.** Postprandial glycemic curves of ten Italian rice genotypes showing a medium Glycemic Index (56 < GI < 70). The graph shows the average blood glucose levels measured in ten healthy volunteers after the ingestion of cooked milled rice, with respect to the glucose solution used as standard (GI = 100).


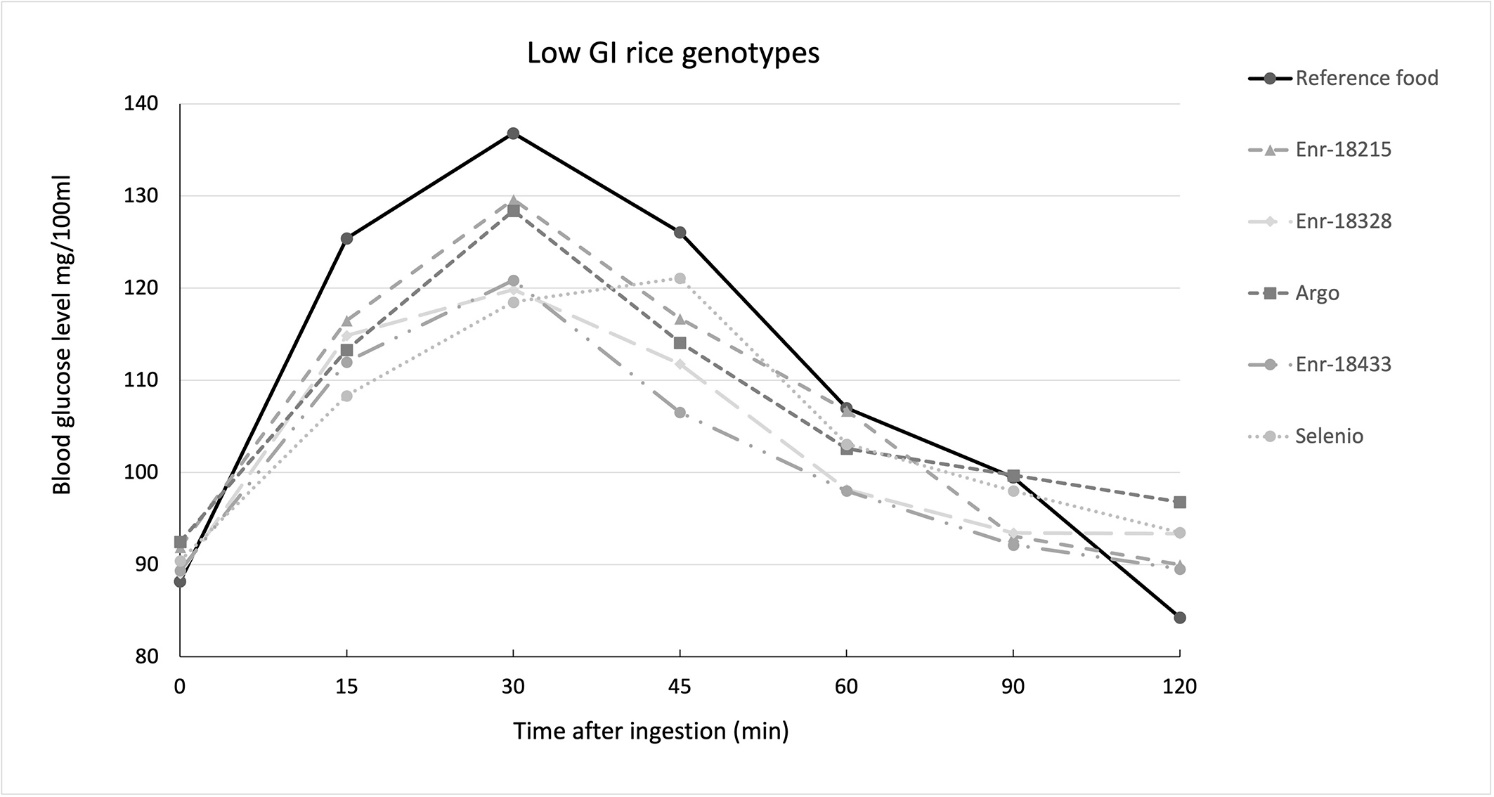


**Supplementary Figure 6.** Postprandial glycemic curves of five Italian rice genotypes showing a low Glycemic Index (< 55). The graph shows the average blood glucose levels measured in ten healthy volunteers after the ingestion of cooked milled rice, with respect to the glucose solution used as standard (GI = 100).
